# From mathematics to medicine: A practical primer on topological data analysis (TDA) and the development of related analytic tools for the functional discovery of latent structure in fMRI data

**DOI:** 10.1371/journal.pone.0255859

**Published:** 2021-08-12

**Authors:** Andrew Salch, Adam Regalski, Hassan Abdallah, Raviteja Suryadevara, Michael J. Catanzaro, Vaibhav A. Diwadkar

**Affiliations:** 1 Department of Mathematics, Wayne State University, Detroit, Michigan, United States of America; 2 Department of Psychiatry & Behavioral Neuroscience, Wayne State University, Detroit, Michigan, United States of America; 3 Department of Mathematics, Iowa State University, Ames, Iowa, United States of America; Museo Storico della Fisica e Centro Studi e Ricerche Enrico Fermi, ITALY

## Abstract

fMRI is the preeminent method for collecting signals from the human brain *in vivo*, for using these signals in the service of functional discovery, and relating these discoveries to anatomical structure. Numerous computational and mathematical techniques have been deployed to extract information from the fMRI signal. Yet, the application of Topological Data Analyses (TDA) remain limited to certain sub-areas such as connectomics (that is, with summarized versions of fMRI data). While connectomics is a natural and important area of application of TDA, applications of TDA in the service of extracting *structure from the* (non-summarized) *fMRI data itself* are heretofore nonexistent. “Structure” within fMRI data is determined by dynamic fluctuations in spatially distributed signals over time, and TDA is well positioned to help researchers better characterize mass dynamics of the signal by rigorously capturing shape within it. To accurately motivate this idea, we a) survey an established method in TDA (“persistent homology”) to reveal and describe how complex structures can be extracted from data sets generally, and b) describe how persistent homology can be applied specifically to fMRI data. We provide explanations for some of the mathematical underpinnings of TDA (with expository figures), building ideas in the following sequence: a) fMRI researchers can and should use TDA to extract structure from their data; b) this extraction serves an important role in the endeavor of functional discovery, and c) TDA approaches can complement other established approaches toward fMRI analyses (for which we provide examples). We also provide detailed applications of TDA to fMRI data collected using established paradigms, and offer our software pipeline for readers interested in emulating our methods. This working overview is both an inter-disciplinary synthesis of ideas (to draw researchers in TDA and fMRI toward each other) and a detailed description of methods that can motivate collaborative research.

## Introduction

Functional magnetic resonance imaging (fMRI) is the preeminent method for a) reaching inferences regarding brain function, b) charactering the organization of macroscopic brain networks and c) understanding the brain’s structure-function relationships [1–3]. fMRI data are high dimensional and complex, and inference is fraught with uncertainty regarding signal origins and their relationship to underlying neurophysiology. Virtually all novel developments in the analyses of fMRI signals rely on *mining the temporal properties of time series data in the service of functional discovery*. Discovery is almost always motivated by the desire to understand structure-function relationships, or to “…understand functional anatomy on the basis of its structural substrates, namely neuronal circuits and connections [4].”

Traditionally, the word “structure” in MRI research denotes *anatomical* structure, and the search for anatomical functional units has remained a vibrant research domain for more than a century [5]; the phrase “function-structure relationships” generally refers to how brain anatomy (structure) constrains or predicts brain responses (function) [6]. However, fMRI data (like other classes of high dimensional data, and particularly neurobiological data) has structure *within* the acquired signal data itself, present in the form of meaningful organization, and symmetric patterns. Topological data analysis (TDA) is optimized to search for specific classes of structure *within* data, and its application to fMRI may provide researchers with concepts and methods that complement existing approaches.

The maturing field of TDA is now at the leading envelope of attempts to understand and characterize *structure within data* [7, 8]. The presumption is that recovery of structure(s) using TDA will reveal important information about the *functional properties* of the system that generated that data. The most significant general idea that we motivate in this paper is that fMRI researchers can and should consider the perspective that a) their *data itself has structure*, and b) that characterizing this structure is an important endeavor in the service of discovery. We buttress this proposal with a working overview of many of the ideas and methods of the rapidly evolving field of TDA, and explain how these ideas are ideally suited for discovering structure in fMRI data. By way of review we present a) the intuitive motivations behind the methods of TDA, b) some ways in which these methods are relevant to fMRI data, and c) some discussion of how TDA complements traditional statistical methods.

Within TDA, we restrict our attention to *persistent homology*, a suite of data-analytic techniques, each of which has the virtue of having essentially no free hyperparameters. While persistent homology is the most heavily researched analytic tool within TDA, others, such as Mapper [9], also exist (though a comprehensive discussion is outside the scope of this paper).

By way of application, we present and implement a practical workflow for applying TDA to reveal dynamics of topological and geometric structure in fMRI data. Our implementation builds on prior conceptual demonstrations and includes code that permits users to emulate our work, as well as examples demonstrating the results of applying this workflow to our own fMRI data.

Throughout, we emphasize that TDA does not replace the many mature statistical approaches for fMRI analysis. Rather, TDA is tuned to asking different questions of the data because its established tools allow for the discovery of patterns and structure in fMRI data that has implications for the study of structure-function relationships in the brain. We attempt this motivation with explanations of the mathematical underpinnings of TDA (accompanied by expository figures), and examples of how TDA can be applied to observed fMRI data.

Some exceptions notwithstanding, fMRI researchers and TD analysts have worked independently of each other. Our position is that fMRI researchers need to be aware of the power of TDA, and TD analysts need to be aware of the complexity and the value of fMRI in neuroscience, and endeavor to see fMRI as an important target for application. This position motivates us to explain abstract mathematical ideas, and their practical application to fMRI data, and our exposition necessarily alternates between these dual motives. To facilitate incremental understanding, we frequently reiterate concepts throughout.

This paper is intended as a *primer* on TDA. It is specific enough to describe a particular workflow for application of a certain topological data-analytic method (“persistence vineyards”) to fMRI, and to describe what kind of organization in fMRI that method is designed to reveal. Given these intentions, we do not attempt *validation* of our method. It would make this paper much more technical and less reader-friendly—and quite a bit longer—to present the ideas in enough detail to offer a careful validation of the method. Detailed and technical exposition of persistence vineyards are widely accessible for interested readers [10].

## Discussion

### Functional discovery in fMRI

Functional discovery is a challenge for any field in which the analyses of overt and observable signals must be used to recover the functionality of a complex system that generated those signals [11]. The problem of functional discovery with fMRI is exacerbated by at least two *physical* limitations of the signal (spatial and temporal resolution), and multiple sources of indeterminacy regarding the signals’ origins [2, 12].

The non-specific origins of the hemodynamic signal constitute a specific challenge. The most proximate drivers of the hemodynamic signal are not neuronal, but metabolic in origin. The polarization and depolarization of cells that underpins neuronal activity generates metabolic demand that effects flow dynamics in the underlying vasculature. [13]. These dynamics in turn leave a magnetic contrast based on the relative concentration of para and di-magnetic oxy (Hb) and deoxy-hemoglobin (dHb) in areas of activated tissue [14, 15]. The most proximate neurophysiological correlates of BOLD are neural oscillations in the low *γ* range [16–20], though this relationship is weak and not universally observed [21, 22]. BOLD appears to be driven by metabolic demands related to peri-synaptic activity [23], rather than neuronal outputs of projection neurons. This rules out some neuronal related inferences that can be drawn from fMRI signals. Despite co-acquisition of hemodynamic and electrophysiological signals, it is widely accepted that the neurophysiological drivers of the signal are multi-faceted. This “many-to-one” mapping from the putative origins of the signal to its observed properties means that fMRI data do not permit *specific* inferences about neuronal process [12].

These limitations are, however, not fundamentally problematic. fMRI signals credibly agglomerate across neuronal processes, and embed within them, *state representations* (at both the local and global scales), of system constituents as they evolve [24]. These state representations readily permit the assessment of macroscopic brain network interactions that can be modelled using an array of mathematical or statistical models [25, 26]. Thus, time series in the rest state can be treated as stochastic fluctuations, that may (or may not) loosely track structural connections between regions [27, 28] and/or that may potentiate networks for cognitive or sensorimotor action [1, 29, 30]. Alternatively, state changes can be experimentally induced, using well-constructed tasks with systematically manipulated experimental conditions within them. Task-based manipulations are highly suited for discovering network interactions because sensorimotor inputs can be treated and modeled as drivers of, or inputs to the system; then, variations in task conditions contextually modulate the intrinsic connectivity of brain networks [31]. These approaches for functional discovery are explicitly motivated by concepts in control theory [32], and how these concepts apply to functional inference in brain networks [33].

More generally, the many-to-one mapping from anatomy to signal is likely to underpin a wide variety of types and scales of organization and patterns. This reinforces the value of studying the structural features in fMRI data on their own terms, and independent of anatomy. TDA provides a powerful set of tools for carrying out such study.

### Relationships with existing work on TDA and fMRI

The earliest allusion to the issue of topology and fMRI data can be found in a compelling review by Worsley [34]. In it, he provided a skillful summary of a) the use of Gaussian Random Fields (a staple representation of all subsequent fMRI analyses), b) the transformation of fMRI (or PET) data in a smoothed Gaussian Random Field into a statistical space, and finally c) how the expected Euler characteristic from a population of t-scores in statistical space can be compared against the observed Euler characteristic in the service of solving the problem of statistical thresholding for conducting multiple comparisons. This paper preceded the development of TDA but was perhaps the first to explicitly link concepts in topology with properties of fMRI and PET data.

Since Worsley’s review, there have been a variety of other studies of brain networks using topological methods, including persistent homology. However, extant applications of TDA to functional neuroimaging data have generally been restricted to quantitative descriptions of the *connectivity* properties of networks. Given any network, a geometric space can be constructed out of the combinatorial properties of the same network and its sub-networks. The topological invariants (a term we will describe in more detail shortly) of this geometric space reflect connectivity properties of the network. In particular, topological properties of the constructed space in high dimensions reflect “higher-order” relationships between sub-networks of the given network. We briefly summarize some of these works. For example, Stolz et al. [35] considered the persistent homology of functional brain *networks* constructed from task-based fMRI data. The work of Anderson et al. [36] explored functional connectivity of resting state fMRI data using persistent homology. Potential pipelines for understanding resting state fMRI using both persistent homology and Mapper have been proposed by Phinyomark et al [37]. Understanding higher order correlations and coherence using simplicial complexes in place of networks has been carried out in [38]. See also [39] for a different approach to TDA and network properties of the fMRI signal, where the relevant network is a “shape graph” consisting of clusters of time indices, rather than clusters of voxels. Giusti et al. [40] study correlations among neurons using persistent homology at a much finer scale compared to the typical fMRI scale used by previous authors. There has also been extensive work by Lee, Chung, and collaborators [41–45]. The study of Lord et. al. [46] investigating the resting state structure of fMRI data using ‘persistent homology scaffolds’ is particularly noteworthy. Other peripheral references include [47, 48]. Rather than apply persistent homology to analyze graphical structures derived from summarized data, we apply these topological methods to fMRI data *directly* without relying on intermediate graphical structure. This is because we are interested in studying the geometry of the fMRI signal itself and drawing inferences from this geometry. To our knowledge, the only study that applied TDA directly to unsummarized fMRI data is [49]. The authors use summary statistics of persistence diagrams in dimension 2 for an age prediction task, as well as vectorized descriptors (via persistence images) to develop cohort brain state trajectories based on age.

Thus, rather than fashioning a geometric space out of combinatorial properties of networks within the brain, we propose using TDA along with the *intrinsic geometry of the fMRI data*, which is already relatively high-dimensional (five-dimensional: three spatial dimensions in real stereotactic space, one time dimension, and one fMRI signal dimension). TDA will allow us to characterize a) the actual fMRI signal itself, b) correlations between signal amplitudes across voxels/regions, c) the *shape* of those activated regions, and of activated pathways and d) the dynamics of how those shapes change over time with changes in the experimental conditions of the task. As far as we are aware, the only paper in the extant literature which applies TDA to understand the geometry of the fMRI signal, along the lines we propose, is Ellis et.al [50]. The authors considered the efficacy of TDA for use in fMRI studies, specifically event-related fMRI, using simulated data. They embedded a geometric loop into an ROI (442 voxels), together with simulated baseline data. Using persistent homology, they could detect the loop, even for relatively low signal change.

We note that our focus tracks closely with the original geometric motivations for the development of the ideas and techniques of algebraic topology: topological methods are used in mathematics to understand the shape and organization of the geometric spaces that arise in differential geometry and differential equations, which in turn arise from mechanical systems in physics and engineering [51, 52]. Our suggestion is that the fMRI signal can be analyzed on the same terms as any other system whose dynamics and geometry we want to understand. Thus, the *topology* of the fMRI signal itself is the avenue for functional discovery.

### A brief primer on topological data analysis

The field of *topological data analysis* (TDA) (see [7, 53, 54] for a general overview of the subject, including some history) can be traced back to the 1990s [55, 56]. Initial studies involved researchers in computer science and mathematics applying tools from algebraic topology, originally intended for the study of geometric problems in pure mathematics, to study shapes and dynamical systems from experimental data.

#### What is topology?

*Topology* is what remains of geometry after forgetting about notions of angle and distance, leaving only considerations of *continuity*, *connectedness*, and especially *continuous functions* and *continuous deformation*.

**Fig 1:**
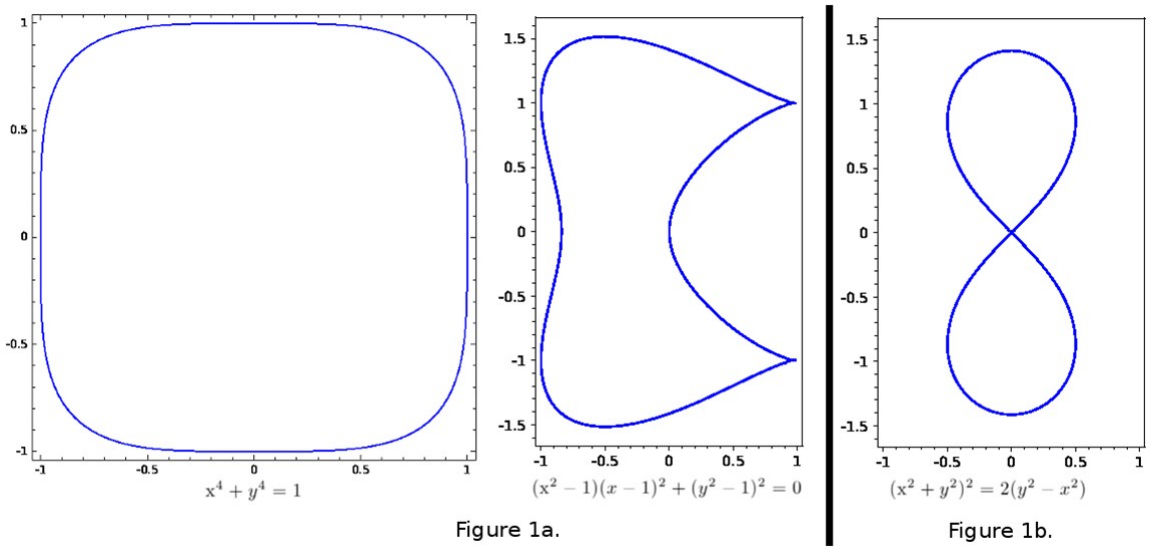
The two curves in (a), are geometrically distinct in many ways, but are homeomorphic, that is, topologically equivalent. Intuitively, each can be continuously deformed into the other without “tearing” or “gluing.” The curve in (b) is, however, not homeomorphic to either of the other curves, because of its node (crossing point) in the center. For curves, these ideas seem straightforward. However, for surfaces and higher-dimensional geometric spaces (which we suggest fMRI-determined structures to be), determining whether two spaces are homeomorphic can be much more involved and less intuitive. In pure mathematics, such determination is a powerful technique in the classification of higher-dimensional geometries.

For a simple example, consider the three curves pictured in Fig 1. *Geometrically*, the curves in Fig 1a are distinct in many ways: 1) The area enclosed by the curve on the left is *convex*, but the area enclosed by the right-hand curve in Fig 1a is not. 2) The arc length (i.e., perimeter; the length of the curve itself) of the left-hand curve is also greater than the arc length of the right-hand curve in Fig 1a. 3) Every point on the left-hand curve has a well-defined tangent line. The same is not true of the right-hand curve in Fig 1a: neither of the *singular points* (the “pinched points”) has a well-defined tangent line.

In terms of geometry the two curves in Fig 1a are clearly distinct. However, *topologically speaking*, they are equivalent. More specifically, they are “*homeomorphic*,” that is, there exists a continuous function, with a continuous inverse function, from one to the other [57]. Intuitively, these functions should be thought of as providing continuous deformations from one curve to the other. We can continuously deform the left-hand curve to the middle curve, without any discontinuous actions like “tearing” or “gluing.” Importantly, since mutually inverse continuous functions map the left-hand curve to the middle curve and vice versa, every genuinely *topological* property of the left curve also holds for the middle curve (and vice versa).

By comparison, the curve in Fig 1b is *not* homeomorphic to either of the curves in Fig 1a. Any curve without a node *cannot* be homeomorphic to a curve with a node. Because the curves in Fig 1a lack any nodes, there can be no homeomorphism from the curve in Fig 1b to either of the two others. These basic examples of curves in the plane generalize readily to higher dimensions such as surfaces, three-dimensional geometries (“three-manifolds”), and *n*-dimensional manifolds for all positive integers *n* (though these generalizations are difficult to visualize).

By finding easy ways to tell that two spaces are *not* homeomorphic, it is possible to show that two geometric spaces are *not* equivalent (e.g., one cannot possibly be obtained from the other by rotations and reflections). For geometric spaces of dimension greater than three, where geometric “intuition” is ineffective, formal topological tools assume importance. These tools help determine if two such geometric spaces could possibly be “equivalent” (e.g., if one space could be obtained from the other by small continuous perturbations, such as “stretching” or “twisting”, but not discontinuous transformations such as “tearing” or “gluing”).

The topological tools employed in persistence are known as *homotopy* and *homology*. Both tools quantify the topological properties of a space: a) how many connected “pieces” does the space consist of, and b) how many loops and voids (and the higher-dimensional analogues of these concepts) does the space have. Throughout this manuscript, we attempt to convey these intuitions (taking some mathematical liberties that blur the distinction between homology and homotopy and preempt technical details from interfering with access to fundamental concepts).

#### What is persistence?

Conventionally, a collection of coordinates in fMRI data form only a discrete, finite set of points in three-dimensional stereotactic space (four-dimensional if signal is included, henceforth referred to as a “stereotactic hyperspace”; the space increases to five-dimensions if time is included as a fifth dimension). To speak of geometry and topology “of the data” in a meaningful way requires more than just a discrete set of isolated points: rather, it requires a choice of “filling in” space around points in stereotactic hyperspace to get a non-discrete space.

**Remark** We will use the standard mathematical symbol $R^{3}$ for the set of triples (*x*, *y*, *z*) of real numbers, which we regard as three-dimensional Euclidean space. By “real stereotactic space,” we mean a copy of 3-dimensional Euclidean space $R^{3}$ in which the discrete voxel coordinates are embedded as points with integer coordinates.

One approach to introducing geometry to a finite set of points is known as the Čech construction. Fix a time index *t* and for each voxel (*x*, *y*, *z*) in stereotactic space, plot the point (*x*, *y*, *z*, *f*(*x*, *y*, *z*, *t*)) in four-dimensional Euclidean space $R^{4}$, where *f*(*x*, *y*, *z*, *t*) represents the BOLD amplitude of the voxel at coordinates (*x*, *y*, *z*) at time index *t*. The result is a finite collection of points in $R^{4}$, often called a “point cloud.” The ambient space $R^{4}$ in which this point cloud is embedded has three spatial dimensions and one signal amplitude dimension, and we regard this version of $R^{4}$ as “real stereotactic hyperspace.” Fill in a four-dimensional ball of radius *r* around each point (*x*, *y*, *z*, *f*(*x*, *y*, *z*, *t*)) of our point cloud. Our main topological space of study is the union of these balls, denoted *C*(*t*, *r*) (for Čech construction), as a subset of real stereotactic hyperspace, where *r* is the radius of the open balls we have filled in around each point in the point cloud, and *t* is the time index.

**Fig 2:**
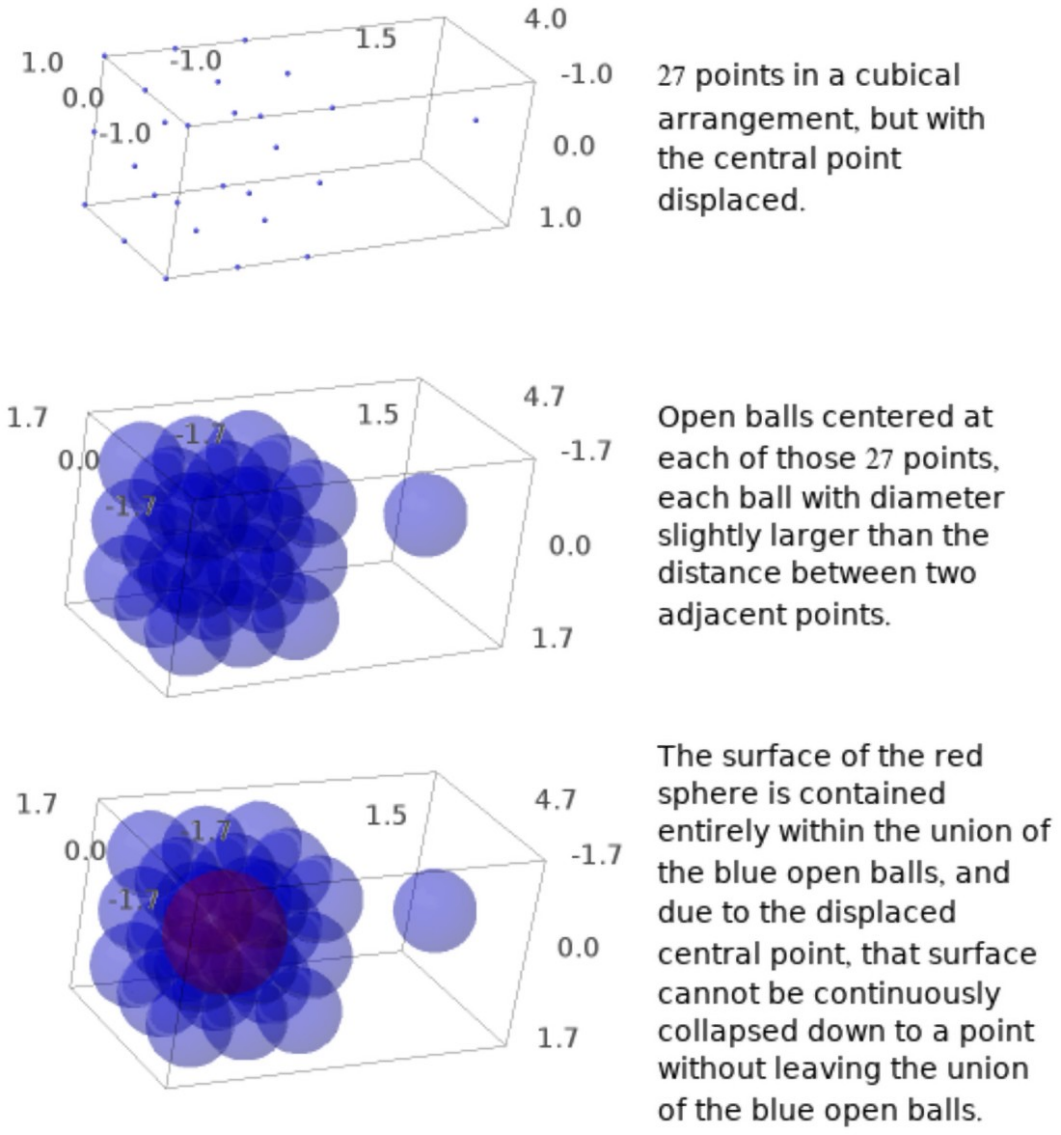
(a) Twenty-seven data points are plotted, in the shape of points at the corners, midpoints of edges, and midpoints of faces of a cube, but the point at the center of the cube is moved several units of distance away. (b) A ball of fixed radius is plotted with its center at that data point. The union of the 26 balls in the left-hand component then appears as a “bumpy” surface of a cube (and is homeomorphic to the surface of a cube). Because the center data point is displaced out of the cube, the ball at the center of the cube is missing from the cube, instead being displaced outside of the cube. Consequently, there is a “hole” in the middle of this “bumpy cube.” (c) The surface of the red sphere is contained entirely inside the union of the balls centered at the data points, but because the center data point is displaced outside of the cube, the red sphere cannot be continuous deformed down to a single point without leaving the union of the balls. This is an example of a sphere, rather than a loop, which cannot be continuously deformed down to a point inside the union of the balls centered at the given data points. Such spheres are closely related to two-dimensional persistent homology. If the radius of the balls is too small, then the balls do not intersect, and so the red sphere cannot be drawn in the union of the balls. On the other hand, if the radius of the balls is too big, then the union of the balls will completely fill the “hole” in the middle of the cube, and the red sphere will be contractible. The range of radii of balls in which the red sphere exists and is contractible is the “persistence” of the red sphere.

If *r* is very small (e.g., smaller than half the edge length of a cubical voxel), then the space *C*(*t*, *r*) will simply be a union of non-intersecting balls, and consequently has no interesting topological features. For example, every continuous loop in *C*(*t*, *r*) has to be contained in a single one of these balls, and so can be contracted down to a single point within that ball. On the other hand, if *r* is sufficiently large, then every ball intersects every other ball, with no gaps left. Thus *C*(*t*, *r*) is convex, and consequently also has no interesting topological features: for example, while continuous loops in *C*(*t*, *r*) can cross between multiple balls, any continuous loop in *C*(*t*, *r*) can be continuously deformed down to a point in *C*(*t*, *r*) using the convexity of *C*(*t*, *r*) (see Fig 2).

But for intermediate values of *r* between the very small and the very large, *C*(*t*, *r*) can take on interesting topological features. Here is an example: consider a ring of voxels of uniformly low signal amplitude, surrounding a cluster of voxels of high signal amplitude, at some time index *t*. If *r* is larger than half the distance between the centers of adjacent voxels, but smaller than half the difference between the high signal and low signal amplitude, then *C*(*t*, *r*) “looks like” a filled-in torus (doughnut shape) together with a filled-in sphere above it, in the dimension which represents the signal amplitude. Topologically, there are two connected components. In the component of lower signal amplitude, there is a continuous loop which cannot be continuously contracted down to a point, namely the loop which wraps around the hole of voxels of high-signal amplitude. See Fig 11 for an example of such a phenomenon in actual fMRI data.

How do we choose the radius *r*? Instead of making arbitrary choices which can bias our results, we should treat the topological properties of *C*(*t*, *r*) *as a function of r*. The crux of persistence is to let *r* vary continuously and track the topological features which *persist* over many (or few) values of *r*. The persistence of a topological feature is the range of *r* values over which it exists (or persists). In this way, we can see pieces merge together, at what radial values are loops formed and die, and reveal how the data are organized in ways that traditional clustering methods cannot.

### On topological features within fMRI data

What are some obvious issues with fMRI related analyses that TDA is optimized to address?

**Fig 3:**
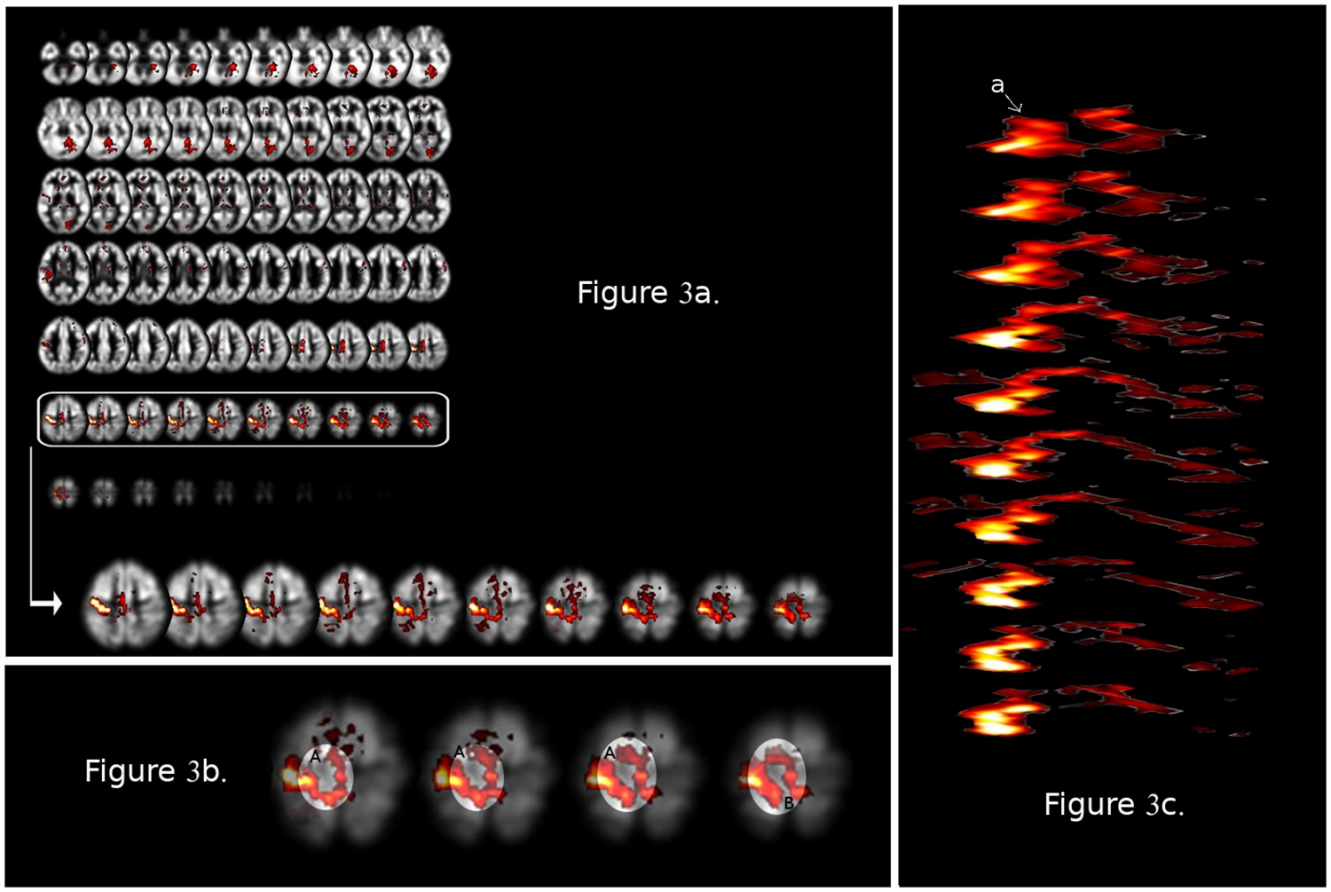
(a) The activation map is adapted from a recent study [63] of the functional organization of motor responses during an inter-hemispheric transfer tasks. The activation map shows activation in contra-lateral motor cortex (for right handed responses). Responses in left primary motor cortex (narrowly defined) are in the inset. Given that this is an activation map, the three-dimensional representation collapses across the dimension of time. The color scale is a statistical representation of signal amplitude. A loop in the lateral and medial aspects of Brodmann Areas 4 and 6 (M1 and Supplementary Motor Area, respectively) suggest a complex topological structure in three-dimensional space the characteristics of which may reflect a hidden structure-function relationship. Notably, activation-based analyses lack the ability to quantify structure in the way that TDA can. This illustrative example can be scaled up to consider the added dimension of time, as TDA approaches can viably identify structure in higher dimensional spaces where the temporal dimension is retained. (b). The four dorsal-most frames from the inset in (a) are shown. As seen, there is a “ring-like” shape within the activated regions. (c) The activated clusters from the cross sections in (a) are extracted from the template, and are stacked in three-dimensional space to form a three-dimensional geometric figure.

**Fig 4:**
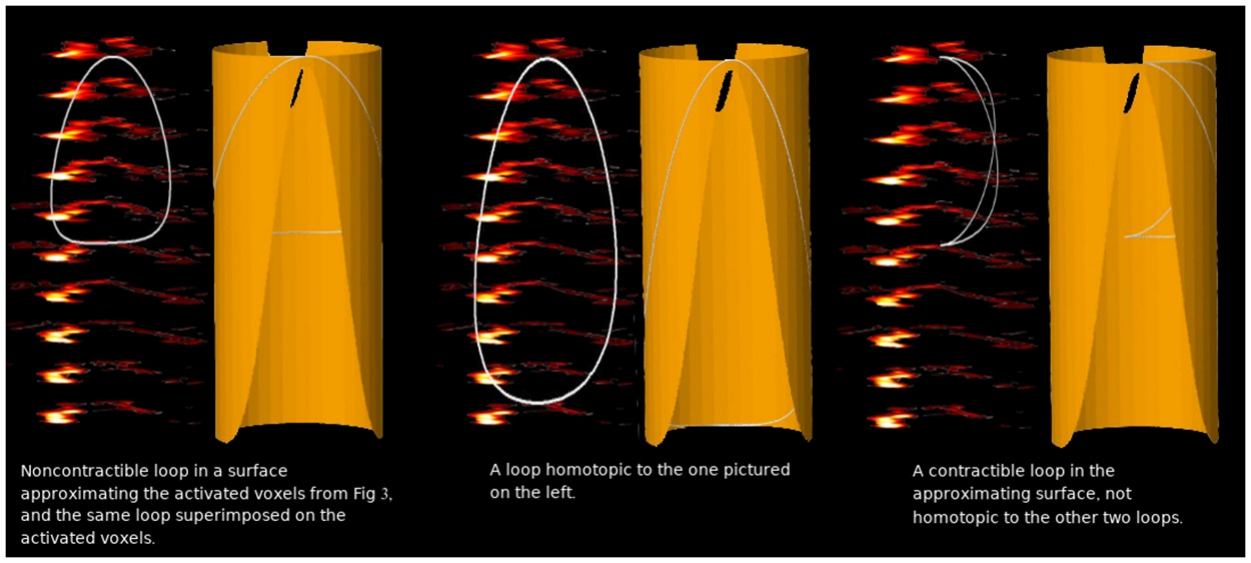
Continuing from Fig 3 see that the two loops on the left are homotopic—they are continuously deformable into one another—in the idealized surface. We present them along with their projections to the fMRI cross-sections which the idealized surface is intended to resemble. The rightmost loop is not homotopic to the two on the left: it is not possible to continuously deform the right-hand loop into either of the two left-hand loops without leaving the idealized surface. In other words, there is no way to continuously deform the right-hand loop into either of the left-hand loops without travelling “too far” in stereotactic space from the activated cluster.

**Fig 5:**
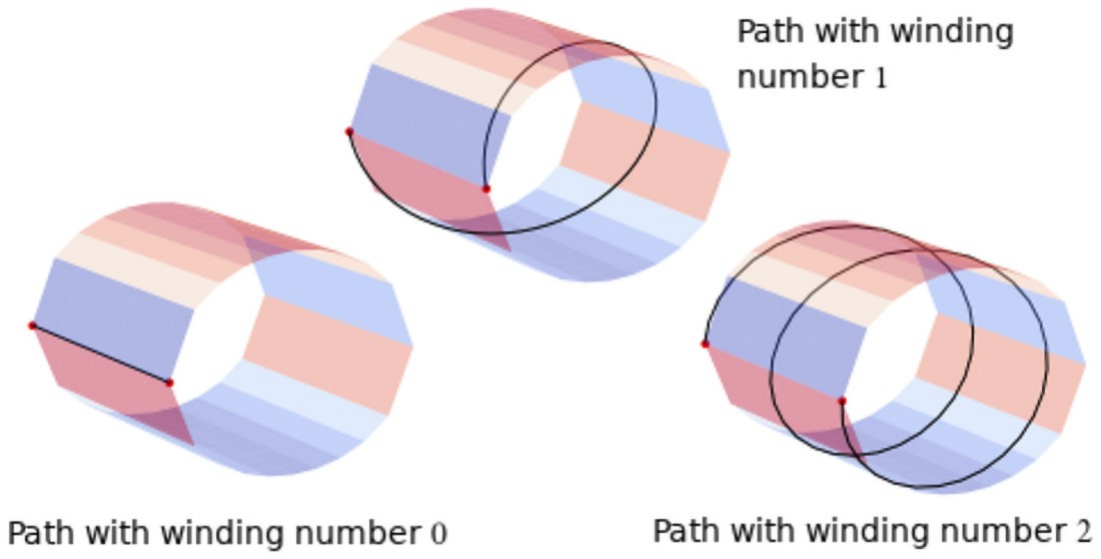
Three different homotopy classes of paths connecting two regions along a cylindrical surface (schematically representing an activated cluster). In general, the homotopy class of a path connecting two points on a cylinder is determined by the winding number of the path, i.e., the number of times the path winds around the central axis of the cylinder. This number is an integer: one can count clockwise winding as positive and counterclockwise as negative, or vice versa. The choice of orientation (clockwise vs. anti-clockwise) gives the two isomorphisms of the fundamental group of a cylindrical surface with the integers under addition.

1. TDA can *reveal structure in fMRI data in a way that is invariant under small perturbations or deformations*, i.e., small anatomical variations from one individual to the next. This property of TDA is important because anatomical variability is a hallmark of brain structures [58, 59] and may confound analyses in stereotactic space. As we explain below, TDA is agnostic regarding assumptions about anatomy, and therefore is not affected by anatomical variations in the way that conventional analyses are; see the section “What isn’t topological about fMRI data,” below, for some discussion of the senses in which persistent homology is and isn’t invariant under anatomical variations.
2. TDA can recover non-linear shape in fMRI data. Generally, because almost all approaches to human brain mapping are concerned with *mapping activation loci to the underlying anatomy, the topology* of fMRI activation profiles is not a subject of inquiry. However, fMRI activation profiles are rife with non-linear “shape” (see Figs 3 and 4 and discussion in subsequent sections, as well as Figs 10 and 11), and TDA can reveal *circumscribed regions within the fMRI data with substantially different signal properties from their surroundings. These might be characterized by distinct geometric (e.g. spherical, toroidal, etc.) organization of the signal with those regions*. Such inquiry may identify functional sub-structures with properties that cut across anatomical regions (see Figs 3 and 4 for an example).
3. TDA can discover properties in the data without a priori information about what the properties should be. Principal component analysis, and dimensional reduction techniques can handle non-linear phenomena, but are generally reliant on knowing the correct change-of-coordinates in order to convert the non-linear phenomenon to a linear one. However, in order to choose the coordinates correctly, one has to already know that the circular shape exists. *This effectively presumes one already knows where to look for non-linear phenomena*, a difficult presumption given our focus on discovery.
4. TDA provides a multiscale approach to the study of data. Even by the standards of complexity associated with biological systems, the brain presents unique challenges with respect to spatial scale, evidenced by poorly understood scaling relationships from the neurotransmitter/molecular levels upwards [60]. Pioneers in the field of biological cybernetics (e.g., Valentino Braitenberg) were aware of these challenges, and advocated for an understanding of the topology of brain anatomy to better understand function [61]. The summaries provided by TDA allow practitioners to study *all scales* (e.g., microscopic to macroscopic) simultaneously. Small values of the aforementioned parameter *r* highlight local structure, whereas larger values reveal structure across the entire region of activation. Hence, small and large scale structure in the data can be examined without *a priori* designation of scale. This notion is the focus of the animated S1 Fig.
5. TDA can recover structure in higher dimensions. As noted, fMRI data by their intrinsic nature are five-dimensional (three spatial dimensions, time, and signal amplitude). Therefore, TDA can reveal the *shapes of regions differentiated by signal, even when those shapes are nonlinear and of dimension three or greater*, without any need for dimensional reduction.
6. Finally, TDA can be adapted in many ways to specific data and problems, and used in parallel with established statistical methods (e.g. activation-based analyses, functional and effective connectivity approaches to network discovery). We explain this further in the section titled “Combining TDA with statistical inference,” below.

We stress that TDA is only one of a set of data-analytic tools, but one that has properties that are complement many other methods and that can make a dent in answer some of the challenging questions in neuroimaging. As do other established methods, it holds the promise of revealing aspects of meaningful organization *in the data*.

#### What is topological about fMRI data?

The search for structure in fMRI data—that is, meaningful, characteristic organization within subsets of fMRI data—need not be linked to spatial *location*. Thus, it may be desirable to look for *structure* in the acquired scans, independent of considerations of anatomy. Individual differences in the functional properties of data may result in the variable topology of functional structures (their shape and/or size), permitting functional discovery in fMRI data that is *invariant under small anatomical perturbations*.

One type of small perturbation is a simple translation in stereotactic space. For example, suppose we take the data of a single fMRI acquisition for one subject, and add a constant to the *y*-coordinate of every voxel. This simple translation would distort the spatial positions of voxels, but this translation *does not change the spatial organization of patterns of relative signal amplitude*, and therefore *does not affect the outcome of topological data analysis*. Because topological methods of data analysis are invariant under such small perturbations, they are resilient to spatial displacement. Accordingly, information about *proximity* (e.g. statements like “paths through low-activation voxels from a given highly-activated voxel cluster to another passes through a spherical low-activation voxel cluster”) can be preserved even as the *correspondence* between voxel coordinates and their mapping to brain anatomy are not necessary for recovering structure *in the data*. However, though TDA methods do not rely on fixed structure-function mapping, and can be conducted in a way that is agnostic of these relationships, all the results from TDA can ultimately be mapped back to anatomy. An example of this is provided in Figs 10 and 11.

#### A motivating example

In this subsection, we show an example of the kind of static structure in fMRI data that non-dynamic methods of TDA are designed to reveal. In subsequent explorations below we extend our analyses to discover structures in the dorsal anterior cingulate that are observed across all conditions of an associative learning paradigm, evidence that it is also possible to discover dynamic structures using TDA (see the Workflow section below).

Here, we focus on of the simplest depiction of observed fMRI data, an activation map produced by averaging signal amplitude over time, as well as by making a choice of threshold value, above which a voxel is considered to be “activated” (see also the previously referenced paper by Worsley). This results in a familiar, easily understandable figure, convenient for our purposes, and allowing us to show that *the methods of TDA work perfectly well without averaging over time and without thresholding*.

In fMRI analyses, it is standard to use tools like Statistical Parametric Mapping [62] to understand how task characteristics induce statistically significant activations in different regions of interest. These activations themselves are organized into clusters of contiguous voxels. A very simple and plausible example of a structure in such data is a *continuous loop* (for brevity, a *loop*). A loop is a genuine *topological* construct, defined in terms of continuous functions, without reference to distance or angle. *Importantly, a continuous loop will be a property inherent in the fMRI data itself*.

Fig 3a shows significant clusters (resulting from a second level random effects analyses) rendered on a mosaic of 70 transverse planes from a recent study from our group [63]. The more superior aspects of the mosaic (sixth row from the top) are highlighted as we will use some observable aspects of this cluster shape in illustrating meaningful properties of TDA.

Mapping the activation peaks to the underlying anatomy is important for functional discovery [64, 65], but an alternative question might be: are there relevant *shapes* in the observed clusters that could be *independent* of the location of the activation peaks. For example, a notable spatial phenomenon in the rightmost slice of the inset is a “ring-like” shape in the activation profile (see Fig 3b). There seem to be two distinct “paths” within the cluster (which has peaks in the primary motor cortex, M1, and the supplementary motor area, SMA), and it is plausible that the cluster is contained within inter-hemispheric white matter tracts. TDA permits the exploration of the topology of this cluster *on its own terms*.

One way to describe the phenomenon indicated in Fig 3a and 3b is that there is a *non-contractible* loop in the observed cluster. That is, we can draw a continuous loop which cannot be continuously deformed (like a rubber-band: it can be stretched, but not torn) down to a single point. This analysis involves some subtlety. For example, the posterior inter-hemispheric connection in the activated regions is not visibly present in the most dorsal cross section (the farthest to the right in the sequence of cross sections in Fig 3a). This is visible as a gap along the hemispheric divide between the activated foci at the posterior extreme of the cluster (labelled “B” in Fig 3b). Instead, a few millimeters further in the ventral direction, we find an interhemispheric connection in the posterior activated regions, which is clearly visible in the third and fourth cross-sections from the right in Fig 3a, and the two leftmost cross sections in Fig 3b. However, there is a more anterior gap, labelled A, in the ventral frames in Fig 3b. Because of these “gaps” in the ring-shaped activated region in each cross-section, we cannot embed a continuous loop in the activated regions in any single cross-section but must instead work in three-dimensions. One can draw a continuous loop entirely within the activated regions indicated in Fig 3a, as follows: a) begin in the right hemisphere, b) cross into the left hemisphere along the posterior interhemispheric crossing indicated in the third cross-section from the right in Fig 3a, and c) then pass back into the right hemisphere along the anterior interhemispheric crossing indicated in the furthest-right cross-section in Fig 3a. This is an example of a non-contractible loop (depicted more clearly in the left-hand subfigure of Fig 4): this loop cannot be continuously contracted down to a single point entirely inside of the activated regions in the fMRI data (for the same reason that a rubber band in the interior of a hollow doughnut, wrapped around the central hole in the doughnut, cannot be contracted down to a point without either tearing the rubber band or leaving the interior of the doughnut).

This non-contractible loop in the activated cluster is only present when using all three spatial dimensions, and not in any single two-dimensional cross-section. The fundamental idea in this simplified example is that reducing the dimensionality of the analyses (from three to two) is insufficient. Recognizing the geometric structure of significantly activated clusters requires working with the full three-dimensional structure. We reiterate that in this highly simplified example, data are collapsed across the dimension of time (as is typical in an activation map).

The activated areas in the cross-sections in Fig 3a–3c can be idealized as cross-sections of a deformed hollow tube, illustrated in Fig 4. A typical technique in topological data analysis is to count such non-contractible loops, *up to continuous deformation*. This relationship of one loop being continuously deformable into another is called “*homotopy*.”

**Fig 6:**
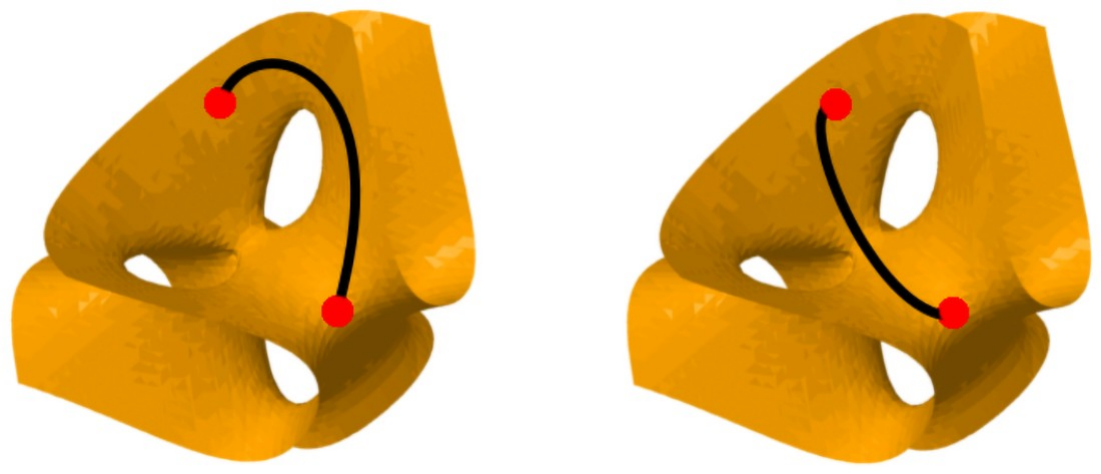
Two different paths connecting two locations within an idealized surface in stereotactic hyperspace (again representing an idealized activated cluster with nontrivial shape). These two paths cannot be continuously deformed into one another without leaving the surface, so these two paths are not homotopic. By tracing one path and then the other, we get a non-contractible loop within the surface of relatively high activation, and consequently a nonzero element in the one-dimensional persistent homology of the fMRI data.

Figs 4–6 are collectively designed to further explicate the concept of homotopy. As noted, any analytic technique for recognizing nonlinear shape in data should be invariant under small deformations. Thus, if this technique is applied to *individual* fMRI activation maps from a given paradigm, we might consistently observe two highly-activated regions that are *always* connected by a tunnel-shaped activated region, even though the *anatomical* location of the topological structures might vary. Thus it may be possible to discover “functional” structures that are common to or cut across individuals. This could make TDA an intriguing tool in the endeavor of discovering individual differences in fMRI data.

#### Homotopy and fMRI

We now attempt to make these mathematical notions rigorous. Assume we have a geometric space *X* (e.g., Figs 4–6). Two paths *p* and *q* are *homotopic* if *p* can be continuously deformed into *q*, inside of the ambient geometric space *X*, without moving the endpoints of the paths. The intuitive mental image is of an elastic string laid down along the path *p*, and pinned down at the two ends so that the ends cannot move. If it is possible to change the path from *p* to *q*, by stretching, twisting, and deforming (*but not breaking*) the string, then the paths *p* and *q* are homotopic. This is illustrated in left-hand and central images in Fig 4. The paths illustrated in Fig 5, on the other hand, are not homotopic to one another.

If two paths *p* and *q* are homotopic, then they are said to be *in the same homotopy class*. Two paths in the same homotopy class can be continuously deformed into each other. From the perspective of topology, these two paths are essentially the same. Another pair of paths which are *not* homotopic to one another is illustrated in Fig 6.

If the two paths are in *different* homotopy classes, then they comprise two genuinely qualitatively *different* connections between their end-points. Examples are illustrated in Figs 5 and 6. For another example, the right-hand path in Fig 4 is also not homotopic to the left-hand and central paths in Fig 4.

One final remark regarding the relationship between paths and loops: if we have two distinct paths *p* and *q*, each from a point *a* to a point *b*, then by traversing *p* to get from *a* to *b*, and then traversing *q* in the reverse direction to get from *b* back to *a*, we obtain a loop within the activated voxels (illustrated in Fig 6). Furthermore, if *p* and *q* are not in the same homotopy class, then the loop is noncontractible. That is, two such paths can be combined to make a loop, and by picking two points on a loop, we can split it to get two paths. This simple observation shows that the calculation of the homotopy classes of non-contractible loops within an activated cluster carries information about the shape of that activated cluster. This is the fundamental connection between paths and loops, and shows how we can characterize the topology of activated pathways via loops.

Thus, given two points in different activated clusters, the mathematical question “*how many homotopy classes of paths within activated voxels connect these two points*?” can become an applied imaging question “*how many genuinely different pathways within activated voxels connect these clusters?*” Framed in this way, the calculation of homotopy classes of paths in fMRI data might resemble a type of “functional tractography” where a path is a functional structure that, if responsive to task-induced fluctuations, might reflect meaningful biological phenomena.

To our knowledge, the search for distinct pathways arising from loops has not yet been used in fMRI analyses. However, this equivalence between a loop and two distinct paths has been fruitful in other biological studies. For example, in studying viral evolution, persistent loops have been shown to give rise to distinct evolutionary pathways involving horizontal and vertical evolution [66], and persistent loops have been shown to give rise to distinct sequences of protein folding and highlight features like compressibility [67–69].

This process of discovery can be achieved with only a minor modification to the above discussion about homotopy classes of paths connecting two clusters: let us denote a significant cluster in fMRI data as *X*. We can consider the set of homotopy classes of loops in *X*. We will refer to this set as the *homology* of *X*. This set has a natural vector space structure (in the sense of linear algebra), and the dimension of this vector space is both explicitly computable via algorithms that are easily implemented, and measures something meaningful about the data. This dimension is, roughly speaking, equal to the number of *holes* in *X* (what we intuitively regard as a “hole” in a geometric space is what a non-contractible loop can be wrapped around).

**Remark** The real situation is that not every element in the first homology of X is a loop. Instead, an element of the first homology of X can be a *linear combination* of loops, and in homology we accept a slightly *weaker* equivalence relation on loops than homotopy. That is, in the first homology of X, two loops can be identified with one another even when one is not continuously deformable into the other. The Hurewicz theorem (see [57] for a textbook treatment) quantifies the relationship homotopy and homology in a rigorous manner. However, a precise treatment of the relationship between homology and homotopy would require a much more serious investment in developing the purely mathematical ideas than is appropriate for this paper. In the interest of restricting the length of the narrative, we are economical with these details here.

With these concepts, we present a more formal depiction of how TDA can be applied to fMRI activation data (Fig 3a). We first stack the activated regions in the cross-sections in three-dimensional space in the way they sit in stereotactic space. A two-dimensional projection of the result (with some rotation and some vertical stretching to make the geometry more visually apparent), is given in Fig 3c. This projection conforms to the cross-sections of a three-dimensional geometric figure in real stereotactic space which looks roughly like a (hollow) tube, but with a split along the side facing the viewer in Fig 4 (this “front side” corresponds to the left anterior side of the cross sections in Fig 3). The split forms near the “top” (dorsal end) of the tube, and widens as we follow it to the “bottom” (ventral end) (Fig 4). We designate the resulting geometric figure (the tube-like figure in Fig 4) as *X*. What is the dimension of the first homology of *X*?

To answer this, we count the number of homotopy classes of loops in *X* in the following way. Choose *a* to be the point indicated with an arrow in Fig 3c. An example loop in *X* would begin at point *a*, and wrap once around the tube-like shape in the clockwise direction. The nature of this wrapping is immaterial so long as the loops wind around the central column once in the clockwise direction. Two possible choices are the loops depicted in the left and in the center in Fig 4.

In fact, we could wrap around the tube *n* times in the clockwise direction, for any integer *n*. A loop which wraps *m* times clockwise around the tube is homotopic to a loop which wraps *n* times around the tube if and only if *m* = *n*. We see consequently that the first homology of *X* is one-dimensional: every homotopy class of loops in *X* is a multiple of the homotopy class of a single loop (wrapping once around *X* in the clockwise direction, or the counterclockwise direction). This is illustrated in Fig 5.

Consequently, the existence of the “hole” in the space *X*, suggested by Fig 4, is reflected algebraically in the first homology of *X* (i.e., the first homology of X is one-dimensional instead of zero-dimensional). If we instead had a space with several such “holes” not connected to one another (as in Fig 6), we would expect to see the first homology of *X* being a vector space of dimension greater than one: roughly, a vector space of dimension equal to the number of “holes” in *X*. As for homotopy, this notion of “homology” is standard in algebraic topology (see [57] for a rigorous construction), and which coincides in many natural cases with a geometrically intuitive sense of the “number of the holes” in a geometric space. Furthermore, the computation of the dimension of the first homology can be done algorithmically, by standard computer libraries such as Ripser, Perseus, Dionysus and GUDHI [70–73], which provide fast and efficient implementations of the computations described here [74].

The concept of homology was originally developed in all dimensions (i.e., not only for loops—one-dimensional “holes”—but also for “voids” and analogous “holes” in higher dimensions) in Poincaré’s influential 1895 “Analysis situs” [75], and has the advantage of being algorithmically computable. Particularly useful for fMRI analysis is a variant called *persistent homology*, a recent (late 20th century) adaptation of homology for application to data arising from empirical measurements. (See [76] for a survey of the subject with historical information on its roots).

Homology in dimension zero counts the number of connected components in a topological space, and persistent homology in dimension zero is a way to describe clustering of points in a data set, e.g. in fMRI data. Classical statistical methods also offer effective ways to describe such clustering, and in particular, single-linkage clustering is precisely persistent homology in degree zero, so this offers little that is new. It is in higher dimensions—e.g. persistent homology in dimension one, which quantitatively reports on loop-shaped organization of points in a data set—that persistent homology can offer us something beyond what well-established statistical methods provide.

Next, we explain how one can go from fMRI data to the homology calculations that describe topological structure *in* the data (when considering the full dimensionality of the data).

#### Five dimensions (space, time, and signal amplitude)

As previously noted, activation based analyses of fMRI data use statistical thresholding to distinguish supra-threshold signal amplitude from sub-threshold amplitude (thus Fig 3a depicts only statistically significant clusters).

Here we instead consider the set of all *quadruples* of real numbers (*x*, *y*, *z*, *f*(*x*, *y*, *z*)), where *f*(*x*, *y*, *z*) is the signal amplitude of the voxel with spatial coordinates *x*, *y*, *z*. Using the usual Euclidean distance in four-dimensional space, two points (*x*_0_, *y*_0_, *z*_0_, *f*(*x*_0_, *y*_0_, *z*_0_)) and (*x*_1_, *y*_1_, *z*_1_, *f*(*x*_1_, *y*_1_,*z*_1_)) are near one another if *x*_0_ and *x*_1_ are very close, and *y*_0_ and *y*_1_ are very close, and *z*_0_ and *z*_1_ are very close, and *f*(*x*_0_,*y*_0_,*z*_0_) and *f*(*x*_1_,*y*_1_,*z*_1_) are very close. In other words, voxels with very different signal amplitudes will be distant from one another in this four-dimensional space, even if these regions are *anatomically* proximate to one another. As in the section on persistence above, we refer to this four-dimensional (three spatial dimensions, one signal amplitude dimension) enrichment of real stereotactic space as “real stereotactic hyperspace.”

The approach of characterizing data in a real stereotactic hyperspace has the natural effect of isolating voxels of very high or very low amplitude, and distinctly higher or distinctly lower than surrounding regions, without the need for statistical thresholding. Moreover, with the added dimension of time, a necessary dimension in all fMRI analyses, the hyperspace *cannot* be visualized. However, TDA is untroubled by the “curse of dimensionality” which affects classical statistical methods [77, 78] and which motivates dimensional-reduction techniques that may lose data and may bias results [79].

In the next sub-section we provide further details regarding *persistent homology*, the tool using which one goes from the actual fMRI data, given by a subset of real stereotactic hyperspace at each time index, to homology calculations which reflect topological structure in that data.

### Persistent homology

The concept of “persistence” addresses each of the following three important questions: a) What definition of “region” is implied in thinking of “activated regions,” b) what is specifically meant by “activated,” and c) given that the methods of TDA are designed to be invariant under small perturbations in the data, are TDA methods robust to outliers (e.g., isolated voxels that might show up as false positives under conventional analyses) [80]?.

A cluster of fMRI coordinates at which the measured signal has higher amplitude than at surrounding coordinates results in a “functional” region of interest, the shape of which can be studied using TDA. However, this may fail to be robust: consider what happens, for example, if there is one voxel with low signal amplitude amidst a cluster of voxels with high signal. The region in real stereotactic space consisting of the voxels of high signal amplitude would form a continuous “shell,” the surface of a sphere, within the highly activated region, wrapped around the one voxel with low signal. The resulting “shell” could not be continuously deformed down to a single point without leaving the regions of high amplitude, because it is wrapped around the one voxel which is not highly activated (as in Fig 2). This is an example of *two-dimensional homology*: rather than considering homotopy classes of loops (i.e., circles) within regions of high amplitude, we are instead considering homotopy classes of spheres within those regions.

We have thus far restricted our descriptions to activated structures (Fig 3), allowing us to more easily describe foundational concepts of TDA. Now, we extend these concepts to incorporate the notion of time as a dimension of interest. Such an extension allows us to demonstrate that the idea of persistence in TDA allows us to work with all the data points in an fMRI acquisition for an experimental participant.

Homology provides a rigorous, quantitative expression of many topological properties of any geometric space. Most importantly for the present situation, *the “hole” made by the cluster of voxels more highly-activated than their surroundings has a quantitative, characteristic effect in persistent homology*. Put simply, homology is a standard way in pure mathematics to rigorously define and measure the existence of “holes” in geometric spaces.

Recall that the Čech construction *C*(*t*, *r*) was defined earlier in this paper, in the “What is persistence?” section. Each non-contractible loop gives rise to non-zero *one-dimensional homology* of the space *C*(*t*, *r*), for appropriate values of *r* depending on the scale of the loop. As a consequence, when we apply this particular method of TDA, *persistent Čech homology*, to fMRI data, what we get is not a listing of the homology of the fMRI data at each time index *t*, but a listing of the homology of the fMRI data at each time index *t and for each radius r*, i.e., the homology of *C*(*t*, *r*). The homology of *C*(*t*, *r*) is trivial for sufficiently small *r* and sufficiently large *r*, since for very small *r*, *C*(*t*, *r*) is a union of isolated, nonintersecting small balls, and for very large *r*, *C*(*t*, *r*) is convex. Consequently, every topological feature of our fMRI data at time index *t* which is visible in the homology has a *birth radius*, the lowest value of *r*, at which that feature is nonzero in the homology of *C*(*t*, *r*), and a *death radius*, the greatest value of *r* at which that feature is nonzero in the homology of *C*(*t*, *r*). The death radius is always at least as large as the birth radius, and the difference is defined to be the persistence of that topological feature:

persistence=deathradius-birthradius.

Persistence is a measure of the geometric (as opposed to topological) scale of a feature in data. Tracking the persistence of topological features in data greatly improves the robustness of persistent homology as a data-analytic method, because *outliers* in data will potentially result in nonzero homology elements with extremely low death radius and consequently extremely low persistence. In the context of fMRI data, a high-persistence topological feature is one which involves voxels separated by large “distances” in the spatial dimension, or in the amplitude dimension, or both. Imagine an abstract data set in which all the points are clustered around the surface of a sphere. Then that sphere is a very high-persistence feature, as it “organizes” all the points in the data set. On the other hand, if you have a tiny “bubble” in the surface of that sphere, that bubble is visible as a low-persistence feature: it “organizes” only a small portion of the data in the set data, and the distances between points in that bubble are small compared to the largest distances between points in the data set (S1 Fig illustrates the idea that topological structure, such as clustering into connected components, can be present at both large and small scales in the same fMRI data).

As is evident, the higher the persistence of an element of the persistent homology in any fMRI data, the more that homology expresses large-scale organization of some substantial part of that data, and just as importantly, it is the high-persistence homology which is least sensitive to artifacts.

We refer the reader to [81] for a discussion of the computational complexity of the calculation of Čech persistent homology. From a practical point of view, the calculation of persistent homology scales very quickly with the size of the data set. Thus persistent homology of whole-brain fMRI data acquired at typical spatial resolutions (e.g. 2 mm^3^) is presently beyond the reach of even a high-performance scientific computing grid. Other variants of persistent homology (e.g. homology of “witness complexes” or cubical complexes) do allow for whole-brain computations, but some summarize whole-brain data in various ways before calculating homology, and can involve additional choices of tuneable hyperparameters. However, restricting analyses to subsets of the data (e.g. only within, say, the anterior cingulate cortex) brings the calculation of persistent homology within reach and (as described in our workflow below), is quite reasonable on a modern laptop computer.

Next, we provide a detailed workflow for computing persistent homology from an experimentally derived data set. We begin with a description of the specific experimental paradigm, and previous studies that have motivated its use. Then we provide a very general diagram describing the workflow that leads to inferences about signal geometry, and correspondingly functional discovery. Finally, we provide source material (including code) used to guide these analyses that (with some modifications) can be deployed in the service of any data set of the reader’s choice.

## Methods

### A workflow for computing persistent homology

This application of the workflow results from fMRI data from a single healthy participant, collected during an associative learning task (all study procedures were approved by Wayne State University’s Institutional Review Board, and the participant gave informed written consent to participate in the fMRI procedures). Several studies from our group have used task-specific fMRI paradigms to study brain network dynamics induced by associative learning and memory [30, 82–88]. Associative learning and memory are characterized by the acquisition of reliably co-occurring environmental contingencies [89]. In a laboratory setting, learning and memory can be efficiently simulated by pairing arbitrary memoranda, the associations between which must be learned in some finite amount of time [90] even without positive or negative contingencies. The complex architecture of human functional brain networks means that these tasks evoke brain responses in both the hippocampus [91] and higher-order control brain regions such as the anterior cingulate cortex (ACC) [92]. The latter region is of particular interest given that it is strongly linked with meta-cognitive processing, sustained attention, and executive control [93], processes that are expected to be in play throughout the course of an associative memory paradigm. Details of the fMRI paradigm can be found elsewhere [30]; in brief, the task alternated between blocks of memory encoding (during which paired memoranda were presented for encoding, 27 s, 3 s/pair), post-encoding consolidation (27 s), cued-retrieval (during which memory for each of the 9 associated pairs was tested, 27 s, 3 s/pair), and post-retrieval consolidation (27 s). Eight iterations were employed (fMRI data were collected with a TR = 3s, 288 images acquired). Preliminary analyses for computing persistent homology are expounded upon in Figs 9–12 (S2 Fig).

**Fig 7:**
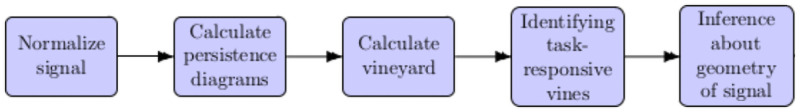
We present our employed workflow used in studying dynamics of persistent homology.

At each time index *t*, let *f*(*x*, *y*, *z*, *t*) be the amplitude of the fMRI signal at time index *t* and voxel coordinates (*x*, *y*, *z*). For each time index *t*, the set of points (*x*, *y*, *z*, *f*(*x*, *y*, *z*, *t*)), ranging across all voxel coordinates (*x*, *y*, *z*) at which we have fMRI data, forms a subset of $R^{4}$ (i.e., real stereotactic hyperspace). Our goal is to use persistent homology to describe topological characteristics of that subset of $R^{4}$ at each moment of time, and then to study the dynamics of those topological characteristics of the signal over time. The steps in our workflow are sketched in Fig 7, with more detail in the text below.

**Fig 8:**
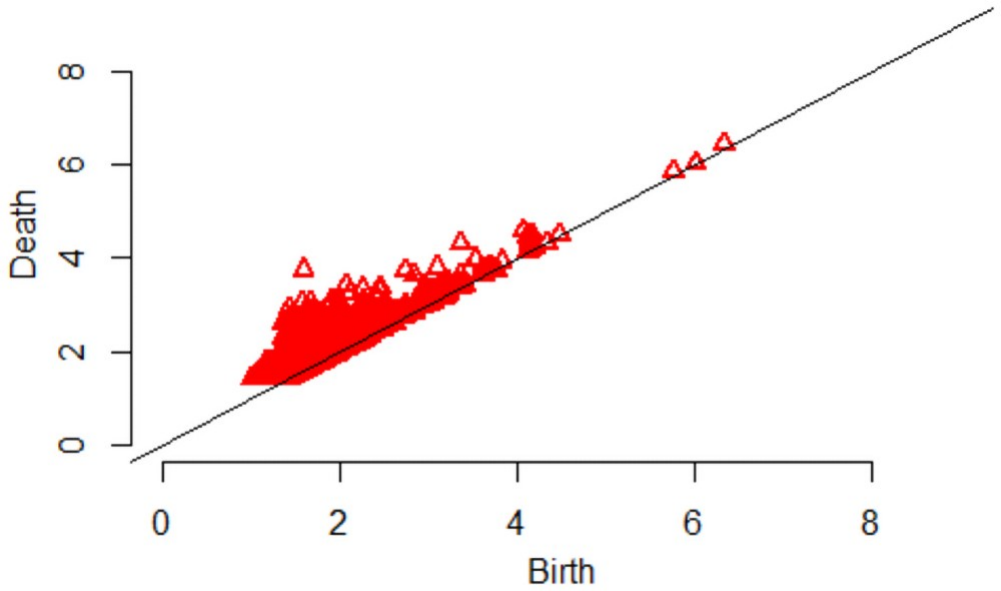
A persistence diagram at one time index.

**Fig 9:**
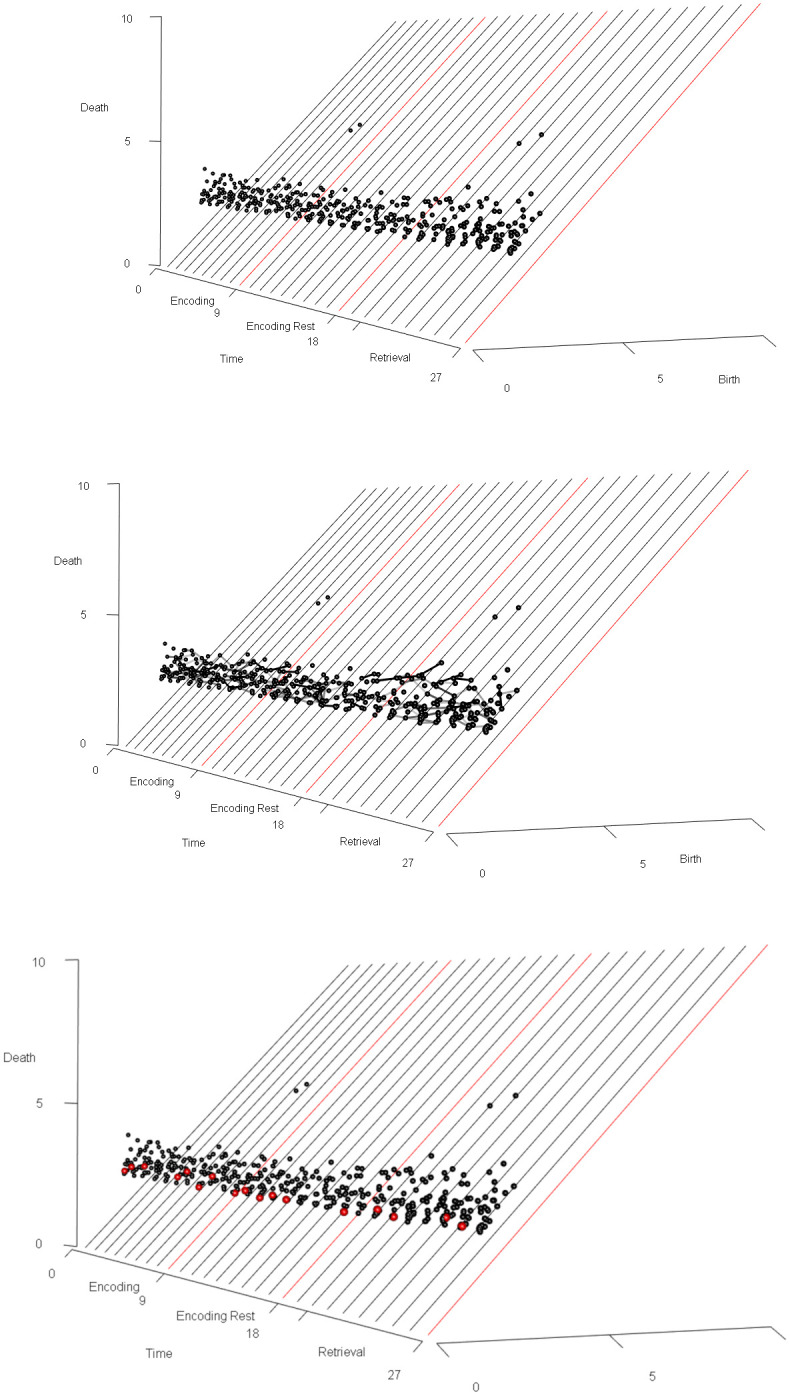
Building from a single time index (in Fig 8), we present persistence diagrams at each of the first 27 time indices in the analyzed fMRI data. The results are stacked to form a three-dimensional image, with time as the third axis (and with birth radius and death radius as the other two axes, consistent with Fig 8). For the sake of visual clarity, we omit features of relatively low persistence from this image. The result is depicted in three complementary views. In the top subfigure, we simply show the stacked persistence diagrams. In the middle subfigure, we present the persistence vineyard, in which the top subfigure is supplemented with line segments indicating the anatomical proximity (i.e., the Hausdorff distance in real stereotactic space) between the representative loops in each homology class. Darker line segments indicate anatomically more proximate loops, while lighter line segments indicate loops which are more anatomically distant from one another. A dark, distinct “vine” represents a loop-shaped topological structure in the fMRI data which is stable over time. The bottom subfigure has red dots indicating a particular vine which our statistical test has identified as being task-correlated, that is, a persistent homology class is present in the vine’s region of stereotactic space at some time indices and not at others, and over the full run of 288 time indices, the presence of the vine at a time index was positively correlated (p<0.01) with that time index being within the memory encoding epoch of the task.

**Normalization of signal.** To scale the dimensions uniformly, the data were normalized so that the scale of the unitless signal amplitude dimension was comparable to the scale of the three spatial dimensions (see Appendix for details about the parameterless normalization procedure for the fMRI signal). We write *f*_*norm*_(*x*, *y*, *z*, *t*) for the normalized signal amplitude at time *t* and stereotactic coordinates (*x*, *y*, *z*).
  The sample results we present below were obtained after applying an anatomical mask to isolate the voxels in the anterior cingulate cortex (ACC).
**Calculation of persistence diagrams.** For each time index *t*, we then calculate the Vietoris-Rips persistent homology, an equivalent but computationally simpler version of Čech persistent homology, of the set of points (*x*, *y*, *z*, *f*_*norm*_(*x*, *y*, *z*, *t*)) in $R^{4}$. For each element ℓ in a basis for the first homology of this set of points (i.e., in Čech terms, each loop in a representative set of noncontractible loops in the union of open balls centered at the points with coordinates (*x*, *y*, *z*, *f*_*norm*_(*x*, *y*, *z*, *t*)) in real stereotactic hyperspace), we record the birth radius of ℓ and the death radius of ℓ, and we plot the set of resulting points, with birth radius on the horizontal axis and death radius on the vertical axis. Due to computational constraints, we implemented a maximum radius threshold of ℓ_*max*_ = 12, effectively truncating the persistence diagram. The resulting plot or *persistence diagram* is shown in Fig 8.
  We clarify that this choice of maximum radius threshold is, comparatively harmless. Increasing the maximum radius threshold in a persistent homology calculation simply results in a more complete persistence diagram, and not the emergence of spurious positive results.
  Each point in the time *t* persistence diagram represents a topological structure present in the fMRI signal at time *t*. Our goal is to understand the *dynamics* of those topological structures, and our next step is motivated by this goal.
**Calculation of the vineyard.** The location of a point in a persistence diagram reflects only the birth radius and death radius of the topological feature that that point represents; it does not reflect the spatial location (in stereotactic space or stereotactic hyperspace) of the “loop” in the data. When studying the dynamics of topological structure, we want to know which topological structures at time *t*+1 are spatially proximate to a given topological structure at time *t*.
  Following this train of thought, we augment the three-dimensional “stacked” sequence of persistence diagrams obtained from Fig 8 with additional visual information: for each point ℓ_*t*_ persistence diagram of the fMRI data at time *t*, and each point ℓ_*t*+1_ in the persistence diagram of the fMRI data at time *t*+1, we draw (in the “stacked” diagram of persistence diagrams) a line segment from ℓ_*t*_ to ℓ_*t*+1_, with shading given by the distance in $R^{3}$ (real stereotactic space, not real stereotactic hyperspace) from a loop representing ℓ_*t*_ to a loop representing ℓ_*t*+1_. The choice of loops representing the homology classes ℓ_*t*_ and ℓ_*t*+1_ are made as in the algorithm given in [94], and as implemented in the software [71]. Consequently, if ℓ_*t*_ is spatially very near ℓ_*t*+1_, a very dark-colored line segment is drawn between the two points in the “stacked” persistence diagrams. If ℓ_*t*_ is spatially very distant from ℓ_*t*+1_, then the line segment connecting the two points is drawn so faintly that it is nearly invisible.
  The resulting three-dimensional diagram is called the *vineyard* of the fMRI data, and it gives a reasonable depiction of dynamical properties of the topological structure inherent in the fMRI data. There are standard references for vineyards as a data-analytic tool [10, 95], although they describe vineyards that differ slightly from ours (e.g. by not using shading on the line segments). A topological structure in the fMRI data which is present at each time index and which remains within a very constrained spatial (in stereotactic space) region results in a very dark, visually distinct “vine” in the vineyards. Structures which respond to an experimental task can result in “intermittent vines”: sequences of points at consecutive time indices connected by dark, visually distinct line segments, but which are not always present (e.g. which are present during only certain epochs of an experiment), or which are not always highly *persistent* during certain epochs of an experiment, resulting in periodic “dips” or “swerves” in the vines.
  Fig 9 displays the vineyard obtained from the ACC-masked data from a patient in our associative learning study. We see that the dynamics of the topological structures present in the data are sophisticated:

- The isolated points high up in the vineyard represent topological structures (i.e., homology classes of loops) in the fMRI data which are sporadic, i.e., present only for a single time index. These structures are of high death radius, but also high birth radius, hence of low persistence. Consequently these topological structures can be regarded as the topological equivalent of noise.
- The darkly-shaded line segments represent topological structures which are present for more than one consecutive time index: two points at consecutive time indices are connected by a darkly-shaded line segment if and only if a loop representing one is proximate, in stereotactic space, to a loop representing the other.
- In the perspective used to display Fig 9, the points which “come out of the page” the most (i.e., those whose death radius most exceeds their birth radius) represent the highest-persistence topological structures. Consequently the “vines” of line segments in Fig 9 which most “come out of the page” represent the large-scale topological structures in the ACC-masked fMRI data, and the starting and stopping of those vines, as we read Fig 9 from left to right, reveal the dynamics of those topological structures.

**Fig 10:**
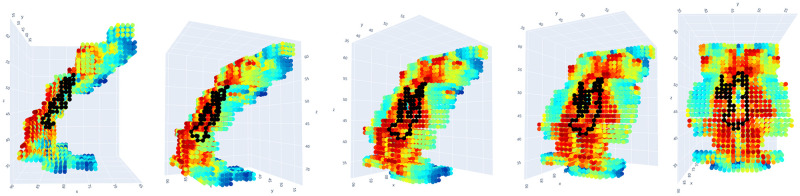
A loop representing the task-correlated persistent vine in the ACC mask for time index 1 is superimposed on a heat map of the fMRI signal amplitude at the same time index. The loop passes through voxels of high signal amplitude and is non-contractible, i.e., cannot be continuously contracted down to a point without passing through voxels of significantly lower amplitude.

**Fig 11:**
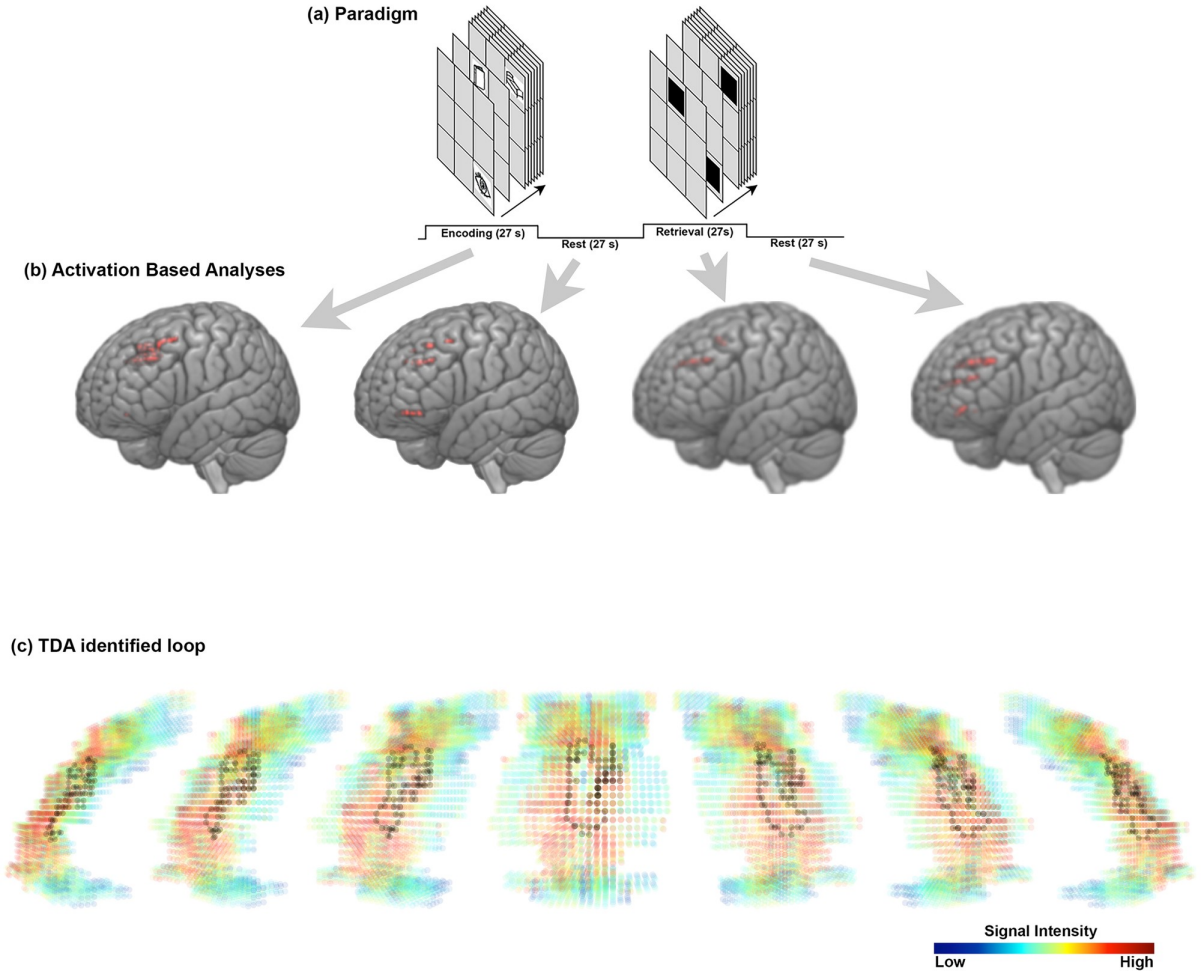
The figure depicts (a) the learning paradigm used to evoke fMRI responses with each of the four conditions clearly depicted, (b) a rendering of the results of activation-based analyses on the anterior cingulate cortex using regressors to represent each of the four conditions (p<.01, corrected), and (c) results of TDA analyses using our documented workflow. The workflow and the statistical test (see text), shows that a non-contractible loop (pictured at time index t = 1) shows significantly more persistence during encoding than during retrieval. Note that the TDA-identified effects in (c) complement the activation-based analyses in (b) in their location in the ACC.

**Fig 12:**
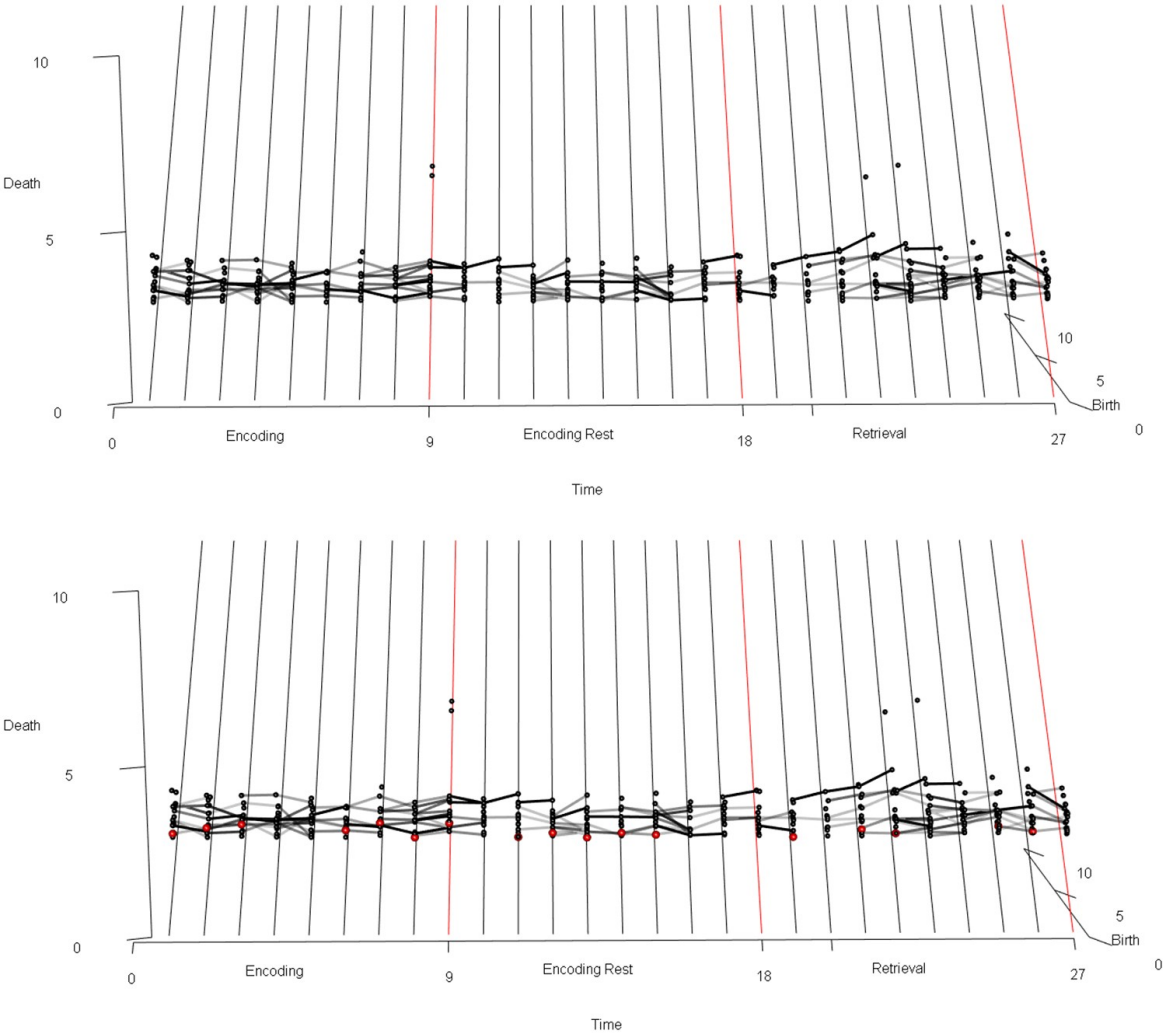
The 27 time indices of the vineyard from Fig 9, from a different perspective. The red dots are members of a statistically identified vine which is intermittent, i.e., not present at every time index. This intermittently-present topological structure forms a “vine” in the sense that it remains within a tightly constrained region of stereotactic space in the ACC. The persistence of this vine at each time index where it is present is correlated with whether that time index is part of the memory encoding epoch of the experimental task. In particular, the vine is statistically significantly more persistent in encoding epochs than in retrieval epochs.

**Identifying task-responsive vines.** Once identified, a vine can be evaluated for covariation with an experimental task. To accomplish this, all persistence diagrams must first be stripped of features that are not members of the vine in question. If there are instances where a persistence diagram does not contain any feature of the vine being evaluated, it is left as empty. The result is a set of persistence diagrams containing *only* features found in a *particular* vine (and with a minimum persistence threshold of 0.8). This new set of persistence diagrams is now amenable to analysis using the statistical inference test described in [97]. It is now possible to determine if the vine exhibits differentiating structure across distinct epochs of an experiment. To begin, the set of persistence diagrams were split based on the original conditions of experiment. The evaluated null hypothesis is that the label assignments based on condition produce two groups no more distinct than if labels were chosen at random. The two groups were compared using a test statistic based on the Wasserstein distance, a metric to compare how similar two persistence diagrams are to each other [97]. The Wasserstein distance captures differences in both persistence and the presence or absence of features between diagrams. Thus, the test statistic compares the groups of persistence diagrams based on both the presence of the vine and its persistence. After the test statistic was computed with the original labels, the labels were then randomly permuted in a manner respecting both the temporal and experimental structure of the task. The test statistic was then computed using these new labels. This process was repeated several thousand times to obtain the distribution of the test statistic under randomly permuted labels, in other words under the null distribution. The test statistic computed under the original labelling is then compared against this null distribution to determine the probability of observing it by chance, i.e. the p-value of our hypothesis test.
  As described above, to identify this task-correlated vine, the persistence diagrams were stripped of all features except those within the given region in stereotactic space, which chosen because of the presence of an unusually persistent homology class in that region at some time indices. The referenced statistical test was then applied to the resulting set of persistence diagrams to evaluate whether the vine’s topological behavior differed between encoding and retrieval epochs. The combination of intermittent presence and varying persistence across epochs elicited a significant p-value (<0.01). Namely, the vine’s behavior is task-responsive.
**Inference about geometry of signal in stereotactic space.** Once a persistent task-responsive “vine” is selected in the vineyard, at each time index *t* at which the vine is present, we select one or more loops within the homology class given by that vine at *t*, and we can plot the voxel locations of those loops. The resultant image represents the loop-shaped structure (which may represent loops of voxels of distinctly higher or lower signal amplitude than surrounding voxels) and is mapped to the anatomy.
  It is illuminating to superimpose loops representing the homology classes in a persistent vine over a heat map of the signal amplitude; Figs 10 and 11 represents this at time index 1 for a homology class in a vine whose presence at each time index is statistically significantly correlated with the experimental task. The data are from the same participant in Figs 8, 9 and 12. As seen, the loop marks out a region of relatively high signal amplitude, wrapped around a region of relatively lower signal amplitude, in the ACC. This differential pattern of signal amplitudes, with its characteristic shape, is what we see as the persistent homology class in the persistent, task-responsive vine in Figs 9 and 12.
  In fact, the sharpness of the difference in amplitudes between these two regions is correlated with the experimental task: during the encoding periods, the loop is more often present than during other periods.

The above workflow can be adapted to specific situations, and we anticipate methodological improvements in some steps. For example, rather than working with a single loop representing each element in first homology, we can carry out our distance calculations (for deciding how darkly to shade each line segment in the vineyard) using many or all loops representing that element in first homology. This would yield a data-analytic method which is more numerically stable, i.e., small changes in the fMRI data would result in comparatively smaller changes in the resulting vineyards.

Our software implementing the above workflow is freely available on a Github repository [98].

#### Robustness

We next discuss the *robustness* of the persistence vineyards produced by the above workflow. Birth-death diagrams are proved to be robust (i.e., small changes in the data set result in small changes in the persistence diagram) in the paper [80].

An *H*_1_ persistence vineyard from fMRI data consists of a collection of birth-death diagrams augmented with line segments that track the proximity, in stereotactic space, of loops representing the homology classes. It has been a recognized issue, since the original paper [10] that introduced persistence vineyards, that these line segments in a persistence vineyard are *not* robust. The issue arises in how loop representatives are chosen, which is not robust in general, and hence, the shading of lines in the vineyard is also not robust. Stated differently, small changes to a data set can yield discontinuous “jumps” in the location of the loop representing a homology class.

One can address these questions of robustness by constructing vineyards using *randomly sampled* representative loops for homology classes. We believe that a serious discussion of this method is too long, too technical, and too purely mathematical (rather than specifically about fMRI) to be within the scope of this paper.

#### What isn’t topological about fMRI data

There presumably are limits to exactly what kinds of “small perturbations” the methods of TDA are invariant under. The paper [80] gives a precise account (with mathematical proofs) of the robustness of persistent homology (the main approach to TDA discussed in the present paper) to perturbations of the data. Further discussion can also be found below, in the “Robustness” section of the present paper.

Furthermore, although the methods of TDA are designed to be invariant under small perturbations, neuroimaging researchers sometimes apply transformations to fMRI data during a preprocessing stage which are *not* invariant under small perturbations. The standard example is the application of a spatial mask to isolate a particular region of interest: of course this step is not invariant under small anatomical variations, and no matter how “perturbation-invariant” a topological data-analytic technique is, it cannot correct for the failure of a preprocessing step, like application of a ROI mask, to be invariant under small anatomical variations.

There are two reasonable, but imperfect responses to the failure of the application of ROI masks to be invariant under small anatomical variations.

1. There may be “boundary effects” due to the placement of the ROI mask which could be mitigated by simply using a permissive mask, i.e., a mask which extends several voxels beyond the desired ROI. This has obvious drawbacks: it is possible that meaningful functional organization from other anatomical regions of the brain other than the ROI will be included in the permissive mask mask (a problem that is also a characteristic of spatial smoothing), so one winds up detecting topological structure in the fMRI data which is not from the anatomical region that one intended to study.
2. Alternatively, one can simply work with whole-brain data. This eliminates the problem of “boundary effects,” but it has two evident difficulties. First, an analysis of whole-brain fMRI data returns a potentially overwhelming amount of information, and one needs a way to spatially localize the topological structures in the fMRI data detected in the analysis, so that one can identify what topological structures in the data are located in particular regions of interest. The “vineyard” approach described in the section “A workflow …” significantly addresses this difficulty.
  The second difficulty with applying TDA to whole-brain data is simply that it is extremely computationally intensive. Recent advances in the design of algorithms for calculation of persistent homology have resulted in enormous improvements [70, 74, 99, 100] in the memory and CPU resources required for TDA on large data sets. The calculation of Cech and Vietoris-Rips persistent homology, the variants we describe in detail, remains largely out of reach for whole-brain fMRI data, but this has been computed for other flavors such as cubical persistence [49].

The last point of the previous paragraph can be rephrased to fit the idea that the *structure* within the fMRI data is *itself* worth studying (a fundamental theme of this paper). If there is some functional phenomenon across subjects which we expect to be reflected in fMRI data, we cannot usually expect this phenomenon to be always located in the same location in stereotactic space because anatomical variability across participants can impact functional responses in unpredictable ways. However, TDA circumvents these issues because the search for functional phenomena in fMRI data is driven by *topology*, rather than an assessment of their location in stereotactic space. Thus, instead of a question like “Is there a cluster of highly-activated voxels in M1 centered at stereotactic coordinates (*x*_1_,*y*_1_,*z*_1_) consistently seen across subjects?” commonly addressed in fMRI studies, TDA is suited to address questions like “Do we see topologically equivalent configurations of high- and low-activation voxels across all subjects during a specific motor task?” The answer to the second question is invariant under most small perturbations in the data (e.g. small anatomical variations). Moreover, the answer has a certain “robustness”, as it is not affected by modest changes to signal amplitude in a single voxel or a relatively small number of voxels. The question instead is about larger-scale organization to the signal amplitude across many voxels (i.e., *structure in the fMRI data*), rather than any single voxel.

### Combining TDA with statistical inference

A natural response to learning about persistent homology is: “How do we know if a topological feature is meaningful?” One answer to this question is that, if a topological feature has markedly higher persistence than others in the same data set, then this feature *should* be investigated, by computing its location within the data set, tracking its change over time, and studying the data points comprising this topological feature. For example, is it a) a very low-signal-amplitude loop surrounded by high-signal-amplitude voxels, b) or the reverse, c) or is the loop composed of two distinct paths connecting a low-signal-amplitude region to a high-signal-amplitude region, d) or some other alternative?

It is valuable to be able to use standard statistical methods to give some quantifiable sense of whether a given topological feature has “markedly higher persistence” than others. A crude approach would be to study the distribution of persistences of topological features in a data set, and to study only the topological features whose persistence is, for example, two standard deviations above the norm within that data set. We refer to [101] for a much more refined approach that uses Monte Carlo methods to arrive at a *p*-value. A recent publicly-available preprint [96] adapts this approach for application to task-based fMRI data, using a multi-level block sampled Monte Carlo test on persistence diagrams to overcome an independence assumption in [101] which limits that paper’s direct applicability to time-series fMRI data.

TDA has also been used in conjunction with statistics and machine learning techniques through various vectorization schemes. However, persistence diagrams do not possess a natural vector space structure amenable to statistical quantities, like mean and variance, so they must be “vectorized” before passed onto statistical learning algorithms. A multitude of authors have proposed various vectorization schemes, known as feature maps, for persistence diagrams to remedy this issue. These include both parametric methods [102, 103], like kernel-based approaches [104, 105], and nonparametric methods [106–108]. All these vectorization tools allow the practitioner to apply statistical learning methods, such as support vector machines, feature selection, neural networks, etc., to persistent homology, and combine the two approaches in a meaningful way.

Using statistics with TDA is important in making inferences about topological structure in fMRI data across many subjects, as well as—as we explain in the “Workflow” section of this paper—in making inferences about correlation of a topological feature with a task. However, we suggest that TDA is a useful and robust method for discovering features in the data from *individual* participants, because of its focus on structure in data. When appropriately applied, this aspect can be valuable in the study of individual differences and/or in clinical applications where individual subject variability can overwhelm the sensitivity of group-based studies.

### Conclusions

This paper straddles the boundaries between a conceptual overview and a methodological demonstration. A pure conceptual overview would be academic, and a methodological demonstration without being preceded by the elucidation of central concepts would be poorly directed. Accordingly, our goal was to provide a working overview of some methods of TDA that can inform the search for the discovery of structure in fMRI signals. To achieve this, we decided to structure this paper in order where we 1) provided information on some of the known underpinnings of the fMRI response, before 2) venturing into a primer on TDA with a focus on the concept of persistence; 3) we then provided some motivations for what topological features might be contained in fMRI data, working through a basic (but limited) example using activation maps; 4) from this limited example, we worked through a detailed explanation of the concept of persistent homology, accentuating our explanations by applying persistent homology to real fMRI data, and 5) using the generated results to attempt to drive home the value of using persistent homology to discover topological structure within the spatio-temporal properties of fMRI data; 6) finally, in concluding the overview, we direct the reader to code used in the computations of persistent homology, in the expectation that our methods can be openly tested on their dataset of choice.

The discovery of complex biological function is acutely difficult, even more so in the case of the brain [109], where generative architectures must be recovered from limited, overt signals [24]. Where does TDA fit in the heavily proliferated mix of methods? We propose that TDA can discover mixed structures in fMRI data that involve both spatial organization in real stereotactic space and variations in intensity of fMRI signal. Such structures may be biologically meaningful, if they are shown to appear in concert with task-induced variations. Because their discovery is based on topology in high dimensions, traditional statistical methods are not optimized to detect them. Thus, TDA can provide a meaningful and unique complement to such established methods.

We hope that our exhaustive approach adequately communicates both why TDA should be used in the service of functional discovery in fMRI, and how it can be used.

### Appendix on parameterless normalization of fMRI data

The normalization process that we describe here, mentioned in the “Workflow” section of our paper, has the merit of being completely parameterless. One of the fundamental reasons to normalize fMRI data is to ensure that the range of values taken by the fMRI signal amplitude is not radically different from the range of values taken by the spatial (x, y, and z) coordinates. With that motivation in focus, we describe the simplest normalization procedure which accomplishes that aim: we do not modify the spatial coordinates of locations in stereotactic space at all, and we modify the fMRI signal amplitude by applying the unique *linear* function such that

- the minimum of the normalized signal amplitudes is equal to the average of the minimum x, minimum y, and minimum z coordinates, and
- the maximum of the normalized signal amplitudes is equal to the average of the maximum x, maximum y, and maximum z coordinates.

Consequently the range of values taken by the normalized signal amplitude is equal to the average of the ranges of values taken by the spatial (x, y, and z) coordinates. As a formula:

$$\begin{array}{c} f_{new}\left(x,y,z,t\right)=\frac{f\left(x,y,z,t\right)-a_{0}}{a_{1}-a_{0}}, \end{array}$$

where

$$\begin{array}{cc} a_{0} & =\frac{\left(\text{min.}\;x\;\text{coord.})+(\text{min.}\;y\;\text{coord.})+(\text{min.}\;z\;\text{coord.}\right)}{3},\;\;\;\text{and}\\ a_{1} & =\frac{\left(\text{max.}\;x\;\text{coord.})+(\text{max.}\;y\;\text{coord.})+(\text{max.}\;z\;\text{coord.}\right)}{3}. \end{array}$$

This normalization process is “canonical” in the sense in which the term is often used in mathematics, that is, there are no free parameters anywhere in this normalization process. We do not know of any reason to believe that ours is the only possible, or the best, parameterless normalization method for fMRI data. However, it does have the merit of being characterized *uniquely* by linearity and its effect on the ranges of normalization values.

It also has the merit that the resulting distances between points in stereotactic hyperspace are invariant under a linear change of spatial coordinate applied to the fMRI data before normalization: that is, whether we configure the fMRI machine to record spatial coordinates using units of millimeters, or of centimeters, or of inches, or of voxel edge lengths, after applying our normalization procedure we get the *same* distances between points in stereotactic hyperspace, and consequently the same results from calculation of persistent homology. This is an important demonstration, because it tells us that the persistent homology of normalized fMRI data is determined by the behavior of the fMRI signal in the physical space being scanned, rather than being partially determined by the way one chooses to *represent* that physical space by a system of units of spatial distance.

One can ask what happens if we modulate the scale of the four coordinates differently, or if we establish the scaling of the coordinate using some other (e.g. non-Euclidean) metric (we thank an anonymous referee for these questions). Applying nonlinear scaling functions, or (more drastically) putting a non-Euclidean metric on stereotactic hyperspace, would lose the information about ratios between spatial distances or differences in signal amplitude, or both, so we use linear scaling functions only. It does not seem meaningful to scale the spatial coordinates differently from one another, as this distorts the natural spatial organization of the anatomy underlying the fMRI signal. One could, in principle, not use the canonical scale described above, and instead “dial” the scale of the fMRI signal amplitude freely up and down:

- The effect of dialing the scale of the fMRI signal very far up is to introduce a great distance in the signal dimension between each point in stereotactic space and its nearest neighbors. The resulting point cloud in stereotactic hyperspace is nearly linear: a cloud of points spaced over an enormous range of signal values, with comparatively only very minor variation in values in the spatial dimensions. All the loop-like organization is lost, as well as voids and higher-dimensional analogues; i.e., the homology *H*_1_,*H*_2_,… becomes trivial, and the resulting vineyards are free of any vines at all.
- The effect of dialing the scale of the fMRI signal very far down is to collapse stereotactic hyperspace down to stereotactic space, i.e., the point cloud is simply a cubical lattice, with no large-scale organization in the form of loops, voids, etc. The resulting *H*_1_,*H*_2_,… has extremely low persistence (e.g. we get many *H*_1_ classes with birth radius equal to half the voxel edge length, and death radius equal to $\sqrt{2}$ times the birth radius; each such *H*_1_ class arises from each 4-tuple of adjacent voxels arranged as the corners of a square). Since the homology is all low-persistence, the resulting vineyard has no high-persistence vines at all.

So artificially “dialing” the fMRI signal scale sufficiently high or sufficiently low yields a negative result when calculating the persistence vineyard.

## Supporting information

S1 Fig Topological organization in fMRI data at multiple scales. Topological organization in data is often present at more than one scale. In this figure, we see both large-scale clustering (three larger clusters when “zoomed out”) and small-scale clustering (nine smaller clusters when “zoomed in”). The persistence of an element of homology (in this case, the zeroth homology, which is a vector space whose dimension essentially counts the number of clusters) quantifies the scale at which the structure described by that element of homology is present.
(GIF)

S2 Fig Interactive vineyard. This HTML file is an interactive rotatable version of the vineyard pictured in Figs 9 and 12 and described in the Workflow section of the paper.
(HTML)

S3 Fig Interactive loop plot. This HTML file is an interactive rotatable version of the plot of the representative loops at t = 1 in the task-correlated persistent vine in the masked fMRI data pictured in Fig 10 and described in the Workflow section of the paper.
(HTML)

S1 Data fMRI data. The included archive of .csv files has one .csv file for each time index. Each .csv file contains a single matrix, which has one column for each of the spatial coordinates x, y, z and one column for the fMRI signal amplitude at voxel (x, y, z) at that time index. This is normalized scan data for a single patient from our study, described in the paper, with an ACC (anterior cingulate cortex) mask applied. This is the data we used, together with our software implementation of our workflow (available at https://github.com/regalski/Wayne-State-TDA), to produce the persistence diagrams, vineyards, and loop locations pictured in the figures and described in the Workflow section of our paper.
(ZIP)

## References

[1] Deco G, Jirsa VK, McIntosh AR. Resting brains never rest: computational insights into potential cognitive architectures. Trends Neurosci. 2013;36(5):268–74. doi: 10.1016/j.tins.2013.03.001 23561718

[2] Logothetis NK. The ins and outs of fMRI signals. Nat Neurosci. 2007;10(10):1230–2. doi: 10.1038/nn1007-1230 17893716

[3] Sui J, Huster R, Yu Q, Segall JM, Calhoun VD. Function-structure associations of the brain: evidence from multimodal connectivity and covariance studies. Neuroimage. 2014;102 Pt 1:11–23. doi: 10.1016/j.neuroimage.2013.09.044 24084066 PMC3969780

[4] Park HJ, Friston K. Structural and functional brain networks: from connections to cognition. Science. 2013;342(6158):1238411. doi: 10.1126/science.1238411 24179229

[5] Amunts K, Zilles K. Architectonic Mapping of the Human Brain beyond Brodmann. Neuron. 2015;88(6):1086–1107. doi: 10.1016/j.neuron.2015.12.001 26687219

[6] Price CJ, Friston KJ. Functional ontologies for cognition: The systematic definition of structure and function. Cogn Neuropsychol. 2005;22(3):262–75. doi: 10.1080/02643290442000095 21038249

[7] Carlsson G. Topology and data. Bulletin of the American Mathematical Society. 2009;46(2):255–308. doi: 10.1090/S0273-0979-09-01249-X

[8] Singh G, Memoli F, Ishkhanov T, Sapiro G, Carlsson G, Ringach DL. Topological analysis of population activity in visual cortex. J Vis. 2008;8(8):11 1–18. doi: 10.1167/8.8.11 PMC2924880 18831634

[9] Singh G, Mémoli F, Carlsson G. Mapper: a topological mapping tool for point cloud data. In: Eurographics symposium on point-based graphics. vol. 102; 2017.

[10] Cohen-Steiner D, Edelsbrunner H, Morozov D. Vines and vineyards by updating persistence in linear time. In: Computational geometry (SCG’06). ACM, New York; 2006. p. 119–126. Available from: https://doi-org.proxy.lib.wayne.edu/10.1145/1137856.1137877.

[11] Erdi P. Complexity explained. Springer Science & Business Media; 2007.

[12] Logothetis NK. What we can do and what we cannot do with fMRI. Nature. 2008;453(7197):869–78. doi: 10.1038/nature06976 18548064

[13] Mangia S, Di Salle F, Garreffa G, Esposito F, Giove F, Cirillo S, et al. Perfusion- and BOLD-based fMRI in the study of a human pathological model for task-related flow reductions. Brain Res Bull. 2004;63(1):1–5. doi: 10.1016/j.brainresbull.2003.10.012 15121233

[14] Buxton RB. Dynamic models of BOLD contrast. Neuroimage. 2012;62(2):953–61. doi: 10.1016/j.neuroimage.2012.01.012 22245339 PMC3545646

[15] Logothetis NK, Wandell BA. Interpreting the BOLD signal. Annu Rev Physiol. 2004;66:735–69. doi: 10.1146/annurev.physiol.66.082602.092845 14977420

[16] Logothetis NK. The neural basis of the blood-oxygen-level-dependent functional magnetic resonance imaging signal. Philos Trans R Soc Lond B Biol Sci. 2002;357(1424):1003–37. doi: 10.1098/rstb.2002.1114 12217171 PMC1693017

[17] Mukamel R, Gelbard H, Arieli A, Hasson U, Fried I, Malach R. Coupling between neuronal firing, field potentials, and FMRI in human auditory cortex. Science. 2005;309(5736):951–4. doi: 10.1126/science.1110913 16081741

[18] Muthukumaraswamy SD, Singh KD. Spatiotemporal frequency tuning of BOLD and gamma band MEG responses compared in primary visual cortex. Neuroimage. 2008;40(4):1552–60. doi: 10.1016/j.neuroimage.2008.01.052 18337125

[19] Niessing J, Ebisch B, Schmidt KE, Niessing M, Singer W, Galuske RA. Hemodynamic signals correlate tightly with synchronized gamma oscillations. Science. 2005;309(5736):948–51. doi: 10.1126/science.1110948 16081740

[20] Sanganahalli BG, Herman P, Rothman DL, Blumenfeld H, Hyder F. Metabolic demands of neural-hemodynamic associated and disassociated areas in brain. J Cereb Blood Flow Metab. 2016;36(10):1695–1707. doi: 10.1177/0271678X16664531 27562867 PMC5076793

[21] Adjamian P, Hadjipapas A, Barnes GR, Hillebrand A, Holliday IE. Induced Gamma activity in primary visual cortex is related to luminance and not color contrast: An MEG study. J Vis. 2008;8(7):4 1–7. doi: 10.1167/8.7.4 19146237

[22] Mullen KT, Dumoulin SO, McMahon KL, de Zubicaray GI, Hess RF. Selectivity of human retinotopic visual cortex to S-cone-opponent, L/M-cone-opponent and achromatic stimulation. Eur J Neurosci. 2007;25(2):491–502. doi: 10.1111/j.1460-9568.2007.05302.x 17284191

[23] Viswanathan A, Freeman RD. Neurometabolic coupling in cerebral cortex reflects synaptic more than spiking activity. Nat Neurosci. 2007;10(10):1308–12. doi: 10.1038/nn1977 17828254

[24] Friston KJ, Li B, Daunizeau J, Stephan KE. Network discovery with DCM. Neuroimage. 2011;56(3):1202–21. doi: 10.1016/j.neuroimage.2010.12.039 21182971 PMC3094760

[25] Passingham RE, Rowe JB, Sakai K. Has brain imaging discovered anything new about how the brain works? Neuroimage. 2013;66:142–50. 23123632 10.1016/j.neuroimage.2012.10.079

[26] Silverstein BH, Bressler SL, Diwadkar VA. Inferring the Dysconnection Syndrome in Schizophrenia: Interpretational Considerations on Methods for the Network Analyses of fMRI Data. Front Psychiatry. 2016;7:132. doi: 10.3389/fpsyt.2016.00132 27536253 PMC4971389

[27] Hermundstad AM, Bassett DS, Brown KS, Aminoff EM, Clewett D, Freeman S, et al. Structural foundations of resting-state and task-based functional connectivity in the human brain. Proc Natl Acad Sci U S A. 2013;110(15):6169–74. doi: 10.1073/pnas.1219562110 23530246 PMC3625268

[28] Logothetis NK, Murayama Y, Augath M, Steffen T, Werner J, Oeltermann A. How not to study spontaneous activity. Neuroimage. 2009;45(4):1080–9. doi: 10.1016/j.neuroimage.2009.01.010 19344685

[29] Diwadkar VA, Asemi A, Burgess A, Chowdury A, Bressler SL. Potentiation of motor sub-networks for motor control but not working memory: Interaction of dACC and SMA revealed by resting-state directed functional connectivity. PLoS One. 2017;12(3):e0172531. doi: 10.1371/journal.pone.0172531 28278267 PMC5344349

[30] Ravishankar M, Morris A, Burgess A, Khatib D, Stanley JA, Diwadkar VA. Cortical-hippocampal functional connectivity during covert consolidation sub-serves associative learning: Evidence for an active “rest” state. Brain Cogn. 2019;131:45–55. doi: 10.1016/j.bandc.2017.10.003 29054542 PMC5927855

[31] Penny WD, Stephan KE, Mechelli A, Friston KJ. Comparing dynamic causal models. Neuroimage. 2004;22(3):1157–72. doi: 10.1016/j.neuroimage.2004.03.026 15219588

[32] Franklin GF, Powell JD, Emami-Naeini A. Feedback control of dynamic systems. Seventh edition. ed. Boston: Pearson; 2015.

[33] Gu S, Pasqualetti F, Cieslak M, Telesford QK, Yu AB, Kahn AE, et al. Controllability of structural brain networks. Nat Commun. 2015;6:8414. doi: 10.1038/ncomms9414 26423222 PMC4600713

[34] Worsley KJ. The geometry of random images. Chance. 1996;9(1):27–40. doi: 10.1080/09332480.1996.10542483

[35] Stolz BJ, Emerson T, Nahkuri S, Porter MA, Harrington HA. Topological Data Analysis of Task-Based fMRI Data from Experiments on Schizophrenia. arXiv:180908504 [math, q-bio]. 2018; arXiv preprint.

[36] Anderson KL, Anderson JS, Palande S, Wang B. Topological Data Analysis of Functional MRI Connectivity in Time and Space Domains. In: Wu G, Rekik I, Schirmer MD, Chung AW, Munsell B, editors. Connectomics in NeuroImaging. Cham: Springer International Publishing; 2018. p. 67–77. 10.1007/978-3-030-00755-3_8 PMC7380262 32715304

[37] Phinyomark A, Ibanez-Marcelo E, Petri G. Resting-State fMRI Functional Connectivity: Big Data Preprocessing Pipelines and Topological Data Analysis. IEEE Transactions on Big Data. 2017;3(4):415–428. doi: 10.1109/TBDATA.2017.2734883

[38] Giusti C, Ghrist R, Bassett DS. Two’s company, three (or more) is a simplex. Journal of Computational Neuroscience. 2016;41(1):1–14. doi: 10.1007/s10827-016-0608-6 27287487 PMC4927616

[39] Saggar M, Sporns O, Gonzalez-Castillo J, Bandettini PA, Carlsson G, Glover G, et al. Towards a new approach to reveal dynamical organization of the brain using topological data analysis. Nat Commun. 2018;9(1):1399. doi: 10.1038/s41467-018-03664-4 29643350 PMC5895632

[40] Giusti C, Pastalkova E, Curto C, Itskov V. Clique topology reveals intrinsic geometric structure in neural correlations. Proceedings of the National Academy of Sciences. 2015;112(44):13455–13460. doi: 10.1073/pnas.1506407112 PMC4640785 26487684

[41] Chung MK, Lee H, DiChristofano A, Ombao H, Solo V. Exact topological inference of the resting-state brain networks in twins. Network Neuroscience. 2019;3(3):674–694. doi: 10.1162/netn_a_00091 31410373 PMC6663192

[42] Lee H, Kang H, Chung MK, Kim BN, Lee DS. Persistent Brain Network Homology From the Perspective of Dendrogram. IEEE Transactions on Medical Imaging. 2012;31(12):2267–2277. doi: 10.1109/TMI.2012.2219590 23008247

[43] Kim H, Hahm J, Lee H, Kang E, Kang H, Lee DS. Brain Networks Engaged in Audiovisual Integration During Speech Perception Revealed by Persistent Homology-Based Network Filtration. Brain Connectivity. 2014;5(4):245–258. doi: 10.1089/brain.2013.0218 PMC4432883 25495216

[44] Lee H, Chung MK, Kang H, Kim BN, Lee DS. Discriminative persistent homology of brain networks. In: 2011 IEEE International Symposium on Biomedical Imaging: From Nano to Macro; 2011. p. 841–844.

[45] Chung MK, Hanson JL, Ye J, Davidson RJ, Pollak SD. Persistent Homology in Sparse Regression and Its Application to Brain Morphometry. IEEE Transactions on Medical Imaging. 2015;34(9):1928–1939. doi: 10.1109/TMI.2015.2416271 25823032 PMC4629505

[46] Lord LD, Expert P, Fernandes HM, Petri G, Van Hartevelt TJ, Vaccarino F, et al. Insights into Brain Architectures from the Homological Scaffolds of Functional Connectivity Networks. Frontiers in Systems Neuroscience. 2016;10. doi: 10.3389/fnsys.2016.00085 27877115 PMC5099524

[47] Ibanez-Marcelo E, Campioni L, Phinyomark A, Petri G, Santarcangelo EL. Topology highlights mesoscopic functional equivalence between imagery and perception: The case of hypnotizability. NeuroImage. 2019;200:437–449. doi: 10.1016/j.neuroimage.2019.06.044 31276797

[48] Ibanez-Marcelo E, Campioni L, Manzoni D, Santarcangelo EL, Petri G. Spectral and topological analyses of the cortical representation of the head position: Does hypnotizability matter? Brain and Behavior. 2019;9(6):e01277. doi: 10.1002/brb3.1277 31001933 PMC6576149

[49] Rieck B, Yates T, Bock C, Borgwardt K, Wolf G, Turk-Browne N, et al. Uncovering the Topology of Time-Varying fMRI Data using Cubical Persistence. arXiv:200607882 [cs, eess, math, q-bio, stat]. 2020;.

[50] Ellis CT, Lesnick M, Henselman-Petrusek G, Keller B, Cohen JD. Feasibility of topological data analysis for event-related fMRI. Network Neuroscience. 2019; p. 1–12. 31410374 10.1162/netn_a_00095 PMC6663178

[51] Arnold V, Khesin B. Topological methods in hydrodynamics. Bull Amer Math Soc. 2000;37:175–181.

[52] Arnold V. Sur la géométrie différentielle des groupes de Lie de dimension infinie et ses applications à l’hydrodynamique des fluides parfaits. In: Annales de l’institut Fourier. vol. 16; 1966. p. 319–361.

[53] Perea JA. A Brief History of Persistence. arXiv:180903624. 2018; arXiv preprint.

[54] Munch E. A user’s guide to topological data analysis. Journal of Learning Analytics. 2017;4(2):47–61. doi: 10.18608/jla.2017.42.6

[55] Frosini P. Measuring shapes by size functions. In: Intelligent Robots and Computer Vision X: Algorithms and Techniques. vol. 1607. International Society for Optics and Photonics; 1992. p. 122–133.

[56] Robins V. Towards computing homology from finite approximations. In: Topology proceedings. vol. 24; 1999. p. 503–532.

[57] Hatcher A. Algebraic Topology. Cambridge University Press; 2002.

[58] Amunts K, Kedo O, Kindler M, Pieperhoff P, Mohlberg H, Shah NJ, et al. Cytoarchitectonic mapping of the human amygdala, hippocampal region and entorhinal cortex: intersubject variability and probability maps. Anat Embryol (Berl). 2005;210(5-6):343–52. doi: 10.1007/s00429-005-0025-5 16208455

[59] Eickhoff SB, Stephan KE, Mohlberg H, Grefkes C, Fink GR, Amunts K, et al. A new SPM toolbox for combining probabilistic cytoarchitectonic maps and functional imaging data. Neuroimage. 2005;25(4):1325–35. doi: 10.1016/j.neuroimage.2004.12.034 15850749

[60] Shulman RG, Rothman DL. Interpreting functional imaging studies in terms of neurotransmitter cycling. Proc Natl Acad Sci U S A. 1998;95(20):11993–8. doi: 10.1073/pnas.95.20.11993 9751778 PMC21753

[61] Negrello M. Valentino Braitenberg: From neuroanatomy to behavior and back. Biol Cybern. 2014;108(5):527–39. doi: 10.1007/s00422-012-0533-3 23483220

[62] Worsley KJ, Marrett S, Neelin P, Vandal AC, Friston KJ, Evans AC. A unified statistical approach for determining significant signals in images of cerebral activation. Hum Brain Mapp. 1996;4(1):58–73. doi: 10.1002/(SICI)1097-0193(1996)4:1<58::AID-HBM4>3.0.CO;2-O 20408186

[63] Diwadkar VA, Bellani M, Chowdury A, Savazzi S, Perlini C, Marinelli V, et al. Activations in gray and white matter are modulated by uni-manual responses during within and inter-hemispheric transfer: effects of response hand and right-handedness. Brain Imaging Behav. 2018;12(4):942–961. doi: 10.1007/s11682-017-9750-7 28808866 PMC5812841

[64] Eickhoff SB, Jbabdi S, Caspers S, Laird AR, Fox PT, Zilles K, et al. Anatomical and functional connectivity of cytoarchitectonic areas within the human parietal operculum. J Neurosci. 2010;30(18):6409–21. doi: 10.1523/JNEUROSCI.5664-09.2010 20445067 PMC4791040

[65] Maldjian JA, Laurienti PJ, Kraft RA, Burdette JH. An automated method for neuroanatomic and cytoarchitectonic atlas-based interrogation of fMRI data sets. Neuroimage. 2003;19(3):1233–9. doi: 10.1016/S1053-8119(03)00169-1 12880848

[66] Chan JM, Carlsson G, Rabadan R. Topology of viral evolution. Proceedings of the National Academy of Sciences. 2013;110(46):18566–18571. doi: 10.1073/pnas.1313480110 PMC3831954 24170857

[67] Xia K, Wei GW. Persistent homology analysis of protein structure, flexibility, and folding: Persistent homology for protein. International Journal for Numerical Methods in Biomedical Engineering. 2014;30(8):814–844. doi: 10.1002/cnm.2655 24902720 PMC4131872

[68] Kovacev-Nikolic V, Bubenik P, Nikolić D, Heo G. Using persistent homology and dynamical distances to analyze protein binding. Statistical applications in genetics and molecular biology. 2016;15(1):19–38. doi: 10.1515/sagmb-2015-0057 26812805

[69] Gameiro M, Hiraoka Y, Izumi S, Kramar M, Mischaikow K, Nanda V. A topological measurement of protein compressibility. Japan Journal of Industrial and Applied Mathematics. 2015;32(1):1–17. doi: 10.1007/s13160-014-0153-5

[70] Bauer U. Ripser: a lean C++ code for the computation of Vietoris–Rips persistence barcodes. 2017; software available at https://github.com/Ripser/ripser.

[71] Morozov D. Dionysus. 2012; software available at http://www.mrzv.org/software/dionysus.

[72] Maria C, Boissonnat JD, Glisse M, Yvinec M. The gudhi library: Simplicial complexes and persistent homology. In: International Congress on Mathematical Software. Springer; 2014. p. 167–174.

[73] Nanda V. Perseus, the Persistent Homology Software. 2019; software available at http://www.sas.upenn.edu/vnanda/perseus.

[74] Otter N, Porter MA, Tillmann U, Grindrod P, Harrington HA. A roadmap for the computation of persistent homology. EPJ Data Science. 2017;6(1):17. doi: 10.1140/epjds/s13688-017-0109-5 32025466 PMC6979512

[75] Poincare H. Analysis situs. Gauthier-Villars; 1895.

[76] Edelsbrunner H, Harer J. Persistent homology–a survey. Contemporary mathematics. 2008;453:257–282. doi: 10.1090/conm/453/08802

[77] Bellman R. Dynamic programming. Science. 1966;153(3731):34–37. doi: 10.1126/science.153.3731.34 17730601

[78] Bellman RE. Adaptive control processes: a guided tour. vol. 2045. Princeton university press; 2015.

[79] Donoho DL. High-dimensional data analysis: The curses and blessings of dimensionality. In: Ams Conference on Math Challenges of the 21st Century; 2000.

[80] Cohen-Steiner D, Edelsbrunner H, Harer J. Stability of persistence diagrams. Discrete Comput Geom. 2007;37(1):103–120. doi: 10.1007/s00454-006-1276-5

[81] Zomorodian A, Carlsson G. Computing persistent homology. Discrete Comput Geom. 2005;33(2):249–274. doi: 10.1007/s00454-004-1146-y

[82] Stanley JA, Burgess A, Khatib D, Ramaseshan K, Arshad M, Wu H, et al. Functional dynamics of hippocampal glutamate during associative learning assessed with in vivo 1H functional magnetic resonance spectroscopy. Neuroimage. 2017;153:189–197. doi: 10.1016/j.neuroimage.2017.03.051 28363835 PMC5498221

[83] Diwadkar VA, Flaugher B, Jones T, Zalányi L, Ujfalussy B, Keshavan MS, et al. Impaired associative learning in schizophrenia: behavioral and computational studies. Cognitive Neurodynamics. 2008;2(3):207. doi: 10.1007/s11571-008-9054-0 19003486 PMC2518754

[84] Diwadkar VA, Bellani M, Ahmed R, Dusi N, Rambaldelli G, Perlini C, et al. Chronological age and its impact on associative learning proficiency and brain structure in middle adulthood. Behavioural Brain Research. 2016;297:329–337. doi: 10.1016/j.bbr.2015.10.016 26462573

[85] Wadehra S, Pruitt P, Murphy ER, Diwadkar VA. Network dysfunction during associative learning in schizophrenia: increased activation, but decreased connectivity: an fMRI study. Schizophrenia research. 2013;148(1-3):38–49. doi: 10.1016/j.schres.2013.05.010 23759649

[86] Woodcock EA, Wadehra S, Diwadkar VA. Network profiles of the dorsal anterior cingulate and dorsal prefrontal cortex in schizophrenia during hippocampal-based associative memory. Frontiers in systems neuroscience. 2016;10:32. doi: 10.3389/fnsys.2016.00032 27092063 PMC4823313

[87] Bányai M, Diwadkar VA, Érdi P. Model-based dynamical analysis of functional disconnection in schizophrenia. Neuroimage. 2011;58(3):870–877. doi: 10.1016/j.neuroimage.2011.06.046 21726653 PMC3221737

[88] Brambilla P, Cerruti S, Bellani M, Perlini C, Ferro A, Marinelli V, et al. Shared impairment in associative learning in schizophrenia and bipolar disorder. Progress in Neuro-Psychopharmacology and Biological Psychiatry. 2011;35(4):1093–1099. doi: 10.1016/j.pnpbp.2011.03.007 21420463

[89] Schultz W, Dickinson A. Neuronal coding of prediction errors. Annual review of neuroscience. 2000;23(1):473–500. doi: 10.1146/annurev.neuro.23.1.473 10845072

[90] Büchel C, Coull J, Friston K. The predictive value of changes in effective connectivity for human learning. Science. 1999;283(5407):1538–1541. doi: 10.1126/science.283.5407.1538 10066177

[91] Ranganath C, Heller A, Cohen MX, Brozinsky CJ, Rissman J. Functional connectivity with the hippocampus during successful memory formation. Hippocampus. 2005;15(8):997–1005. doi: 10.1002/hipo.20141 16281291

[92] Woodcock EA, White R, Diwadkar VA. The dorsal prefrontal and dorsal anterior cingulate cortices exert complementary network signatures during encoding and retrieval in associative memory. Behavioural brain research. 2015;290:152–160. doi: 10.1016/j.bbr.2015.04.050 25960314

[93] Paus T. Primate anterior cingulate cortex: where motor control, drive and cognition interface. Nature reviews neuroscience. 2001;2(6):417. doi: 10.1038/35077500 11389475

[94] Delfinado CJA, Edelsbrunner H. An incremental algorithm for Betti numbers of simplicial complexes on the 3-sphere. vol. 12; 1995. p. 771–784. Available from: https://doi-org.proxy.lib.wayne.edu/10.1016/0167-8396(95)00016-Y.

[95] Munch E. Applications of Persistent Homology to Time Varying Systems [PhD in Mathematics]. Duke University. Durham, NC; 2013.

[96] Abdallah H, Regalski A, Kang MB, Berishaj M, Nnadi N, Chowdury A, et al. Statistical Inference for Persistent Homology applied to fMRI. available in preprint form. 2020; https://github.com/hassan-abdallah/.pdf.

[97] Mileyko Y, Mukherjee S, Harer J. Probability measures on the space of persistence diagrams. Inverse Problems. 2011;27(12):124007, 22. doi: 10.1088/0266-5611/27/12/124007

[98] Abdallah H, Regalski A. Wayne State TDA Github repository. 2020; https://github.com/regalski/Wayne-State-TDA.

[99] Bauer U, Kerber M, Reininghaus J. Distributed Computation of Persistent Homology. In: 2014 Proceedings of the Sixteenth Workshop on Algorithm Engineering and Experiments (ALENEX). Proceedings. Society for Industrial and Applied Mathematics; 2013. p. 31–38.

[100] The GUDHI Project. GUDHI User and Reference Manual. 3.4.1 ed. GUDHI Editorial Board; 2021. Available from: https://gudhi.inria.fr/doc/3.4.1/.

[101] Robinson A, Turner K. Hypothesis Testing for Topological Data Analysis; 2013.

[102] Adams H, Emerson T, Kirby M, Neville R, Peterson C, Shipman P, et al. Persistence images: A stable vector representation of persistent homology. The Journal of Machine Learning Research. 2017;18(1):218–252.

[103] Carrière M, Oudot SY, Ovsjanikov M. Stable topological signatures for points on 3d shapes. In: Computer Graphics Forum. vol. 34. Wiley Online Library; 2015. p. 1–12.

[104] Reininghaus J, Huber S, Bauer U, Kwitt R. A stable multi-scale kernel for topological machine learning. In: Proceedings of the IEEE conference on computer vision and pattern recognition; 2015. p. 4741–4748.

[105] Kusano G, Hiraoka Y, Fukumizu K. Persistence weighted Gaussian kernel for topological data analysis. In: International Conference on Machine Learning; 2016. p. 2004–2013.

[106] Bubenik P. Statistical topological data analysis using persistence landscapes. The Journal of Machine Learning Research. 2015;16(1):77–102.

[107] Chazal F, Fasy BT, Lecci F, Rinaldo A, Wasserman L. Stochastic convergence of persistence landscapes and silhouettes. Journal of Computational Geometry. 2015;6(2):140–161.

[108] Robins V, Turner K. Principal component analysis of persistent homology rank functions with case studies of spatial point patterns, sphere packing and colloids. Physica D: Nonlinear Phenomena. 2016;334:99–117. doi: 10.1016/j.physd.2016.03.007

[109] Richards BA, Lillicrap TP, Beaudoin P, Bengio Y, Bogacz R, Christensen A, et al. A deep learning framework for neuroscience. Nature neuroscience. 2019;22(11):1761–1770. doi: 10.1038/s41593-019-0520-2 31659335 PMC7115933

