# Supplementary figures and images for: From mathematics to medicine: A practical primer on topological data analysis (TDA) and the development of related analytic tools for the functional discovery of latent structure in fMRI data
Source: PLoS One. 2021 Aug 12;16(8):e0255859. doi: 10.1371/journal.pone.0255859 (PMC8360597; doi:10.1371/journal.pone.0255859)

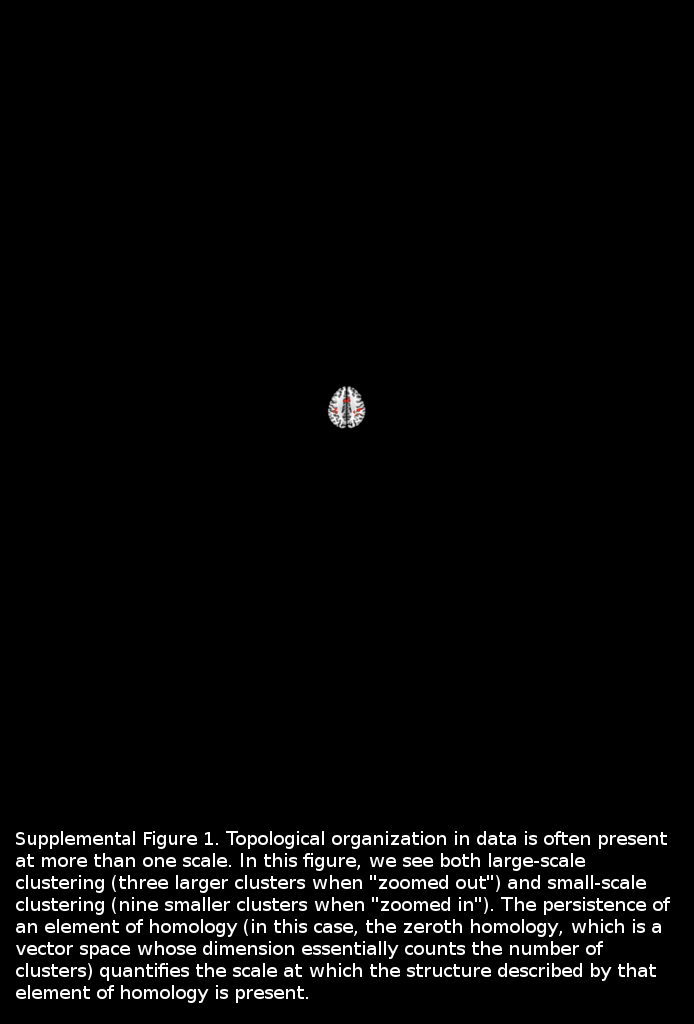

Supplement: S1 Fig — Topological organization in data is often present at more than one scale. In this figure, we see both large-scale clustering (three larger clusters when “zoomed out”) and small-scale clustering (nine smaller clusters when “zoomed in”). The persistence of an element of homology (in this case, the zeroth homology, which is a vector space whose dimension essentially counts the number of clusters) quantifies the scale at which the structure described by that element of homology is present. (GIF) [file pone.0255859.s001.gif]
