# Supplementary material for: From mathematics to medicine: A practical primer on topological data analysis (TDA) and the development of related analytic tools for the functional discovery of latent structure in fMRI data
Source: PLoS One. 2021 Aug 12;16(8):e0255859. doi: 10.1371/journal.pone.0255859 (PMC8360597; doi:10.1371/journal.pone.0255859)
Supplement: S2 Fig — This HTML file is an interactive rotatable version of the vineyard pictured in Figs 9 and 12 and described in the Workflow section of the paper. (HTML) [file pone.0255859.s002.html]

rglWebGL
